# Supplementary material for: Blocking cancer-fibroblast mutualism inhibits proliferation of endocrine therapy resistant breast cancer
Source: Mol Syst Biol. 2025 May 8;21(7):825–55. doi: 10.1038/s44320-025-00104-6 (PMC12222798; doi:10.1038/s44320-025-00104-6)
Supplement: Supplementary file 1 — Appendix [file 44320_2025_104_MOESM1_ESM.pdf]

Appendix: Blocking cancer-fibroblast mutualism inhibits proliferation of endocrine therapy resistant breast cancer.

Authors: Jason I. Griffiths[1,2*,+], Feng Chi[1+], Elena Farmaki[1], Eric F. Medina[1], Patrick A. Cosgrove[1], Kimya L. Karimi[1], Jinfeng Chen[1], Vince K. Grolmusz[1], Frederick R. Adler[2,3], Qamar J. Khan[4], Aritro Nath[1], Jeffrey T. Chang[5], Andrea H. Bild[1*]

Affiliations:
1. Department of Medical Oncology & Therapeutics, City of Hope National Medical Center, 1500 East Duarte Road, Duarte, CA, 91010, USA.

2. Department of Mathematics, University of Utah 155 South 1400 East, Salt Lake City, UT, 84112, USA.

3. School of Biological Sciences, University of Utah 257 South 1400 East, Salt Lake City, UT, 84112, USA.

4. Division of Medical Oncology, Department of Internal Medicine, The University of Kansas Medical Center, Kansas City, KS, 66160, USA.

5. Department of Integrative Biology and Pharmacology, School of Medicine, School of Biomedical Informatics, UT Health Science Center at Houston, Houston, TX, 77030, USA.

+ Co-first authors

* To whom correspondence should be addressed: Jason Griffiths (jasonigriff@gmail.com) and Andrea Bild (abild@coh.org).

Table of Content

Appendix Figure S1 ……… 2
Appendix Figure S2 ……… 3
Appendix Figure S3 ……… 4
Appendix Figure S4 ……… 5

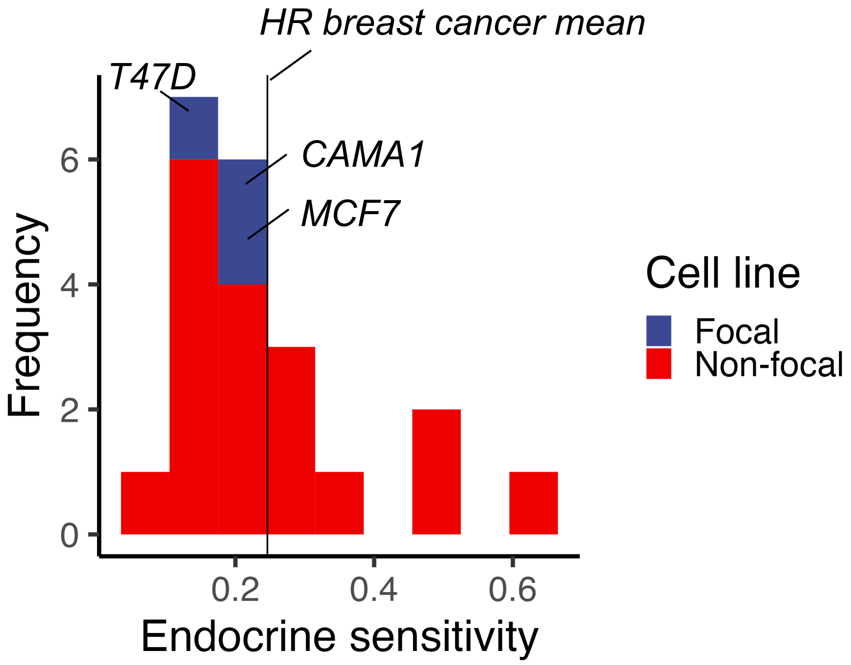

Appendix Figure S1) Endocrine therapy resistance of in vitro cancer cell lines. Histogram of the endocrine sensitivity of the breast cancer cell lines used within this study (blue: focal cell lines= CAMA-1, MCF-7 and T47D) compared to other hormone receptor-positive breast cancer cell lines (non-focal lineages: red). To measure endocrine sensitivity, we utilized drug dose-response data analyzed and published by (Hafner *et al*, 2017). They reported growth rate (GR) based, proliferation-corrected, endocrine sensitivity for 71 breast cancer cell lines, of which we examined 21 hormone receptor-positive luminal subtype cancer cell lines. Endocrine sensitivity measured by GR[AOC] ('area over the curve') to assess the integrated effect of (Z)-4-Hydroxytamoxifen across doses. A value of 0 indicates no drug effect across the full dose-response range. Vertical line indicates mean endocrine sensitivity.

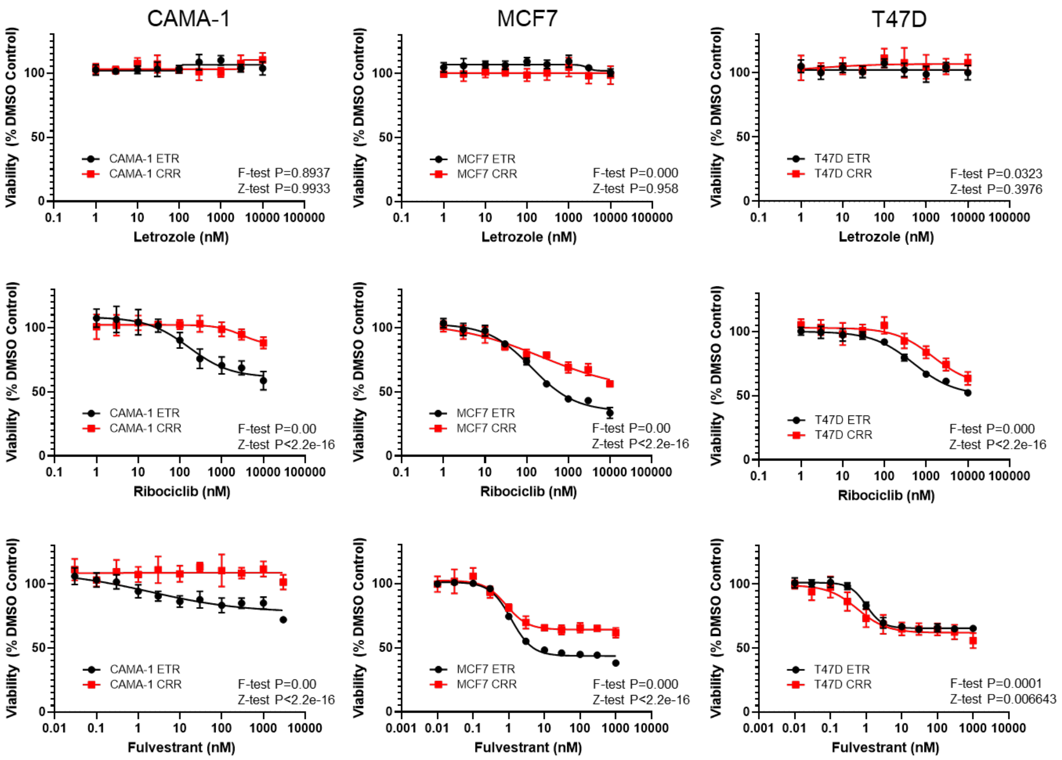

Appendix Figure S2) Confirmation of endocrine and cell cycle therapy resistance of experimentally evolved cancer cell lines. Dose-response curves of viability of CAMA-1, MCF-7 and T47D endocrine therapy resistant (ETR: black) and combination ribociclib resistant (CRR: red) ER+ breast cancer cell lines under letrozole, ribociclib or fulvestrant treatment. Cells were treated with increasing concentration of drug for 4 days and viability was measured using CellTiter-Glo Chemiluminescent kit. Data represents % viable cells compared with DMSO control treatment for each cell line. Data represents % viable cells compared with DMSO control treatment for each cell line and are shown as average of four replicates ± SD. Dose-response curves were generated using GraphPad Prism 9.3.1 software. P-values for the difference in the dose response curves between cell lines were generated using a 4-parameter log-logistic dose response model and F-statistic. P-values for significant difference in ED50 values between cell lines were calculated by Z-test.

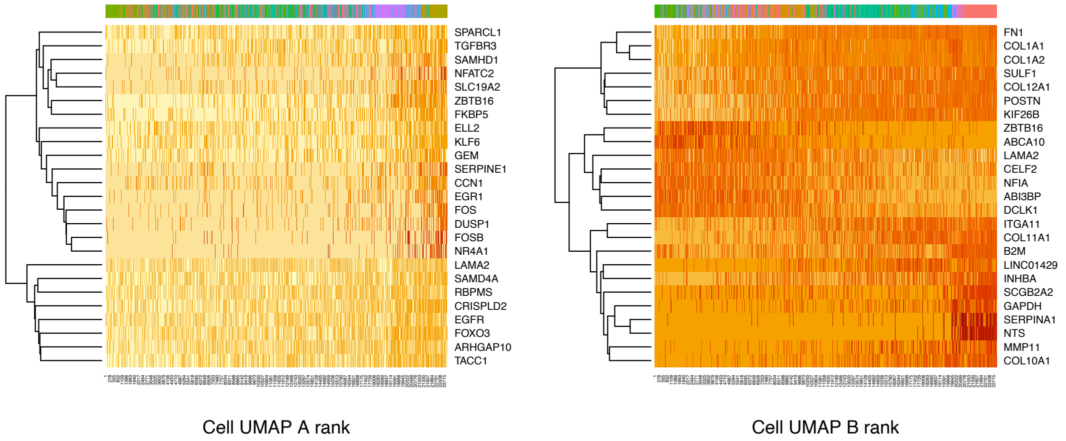

Appendix Figure S3) Trajectory-based differential expression analysis results identify the core transcriptional program defining fibroblast differentiation across the UMAP landscape. Genes dynamically expressed across UMAP A axis (left panel) and UMAP B axis (right panel) were identified using a general additive model to test the potentially nonlinear relationships between scaled gene expression (log(1+CPM)) and UMAP coordinates. Heatmap shows the single cell RNAseq gene expression of the top 25 most dynamically changing genes (rows; grouped by hierarchical clustering) that typically showed monotonically increasing or decreasing expression along the UMAP axes. Columns represent single fibroblast cells that are ordered by rank order along a UMAP axis (annotation bars above indicates fibroblast subpopulation cluster annotation obtained from gaussian mixture model clustering with cluster number determined using Bayesian information criteria). Darker+ redder tile coloration signifies higher expression. Genes dynamically changing along UMAP A include EGFR expression, various downstream EGFR response transcription factors (FOS, FOXO3, FOSB, DUSP1, KLF6, NR4A1). Genes dynamically increasing along UMAP B are involved in extracellular matrix formation (collogens: COL1A1, COL1A2, COL12A1, COL11A1, COL10A1; fibronectin: FN1; ITGA11), extracellular matrix protein secretion (POSTN, B2M, MMP11), mesenchymal fibroblast differentiation (INHBA, KIF26B). Genes dynamically downregulated along UMAP B are involved in inhibition of stromal differentiation (NFIA, DCLK1) and regulation of proliferation (ABI3BP, CELF2).

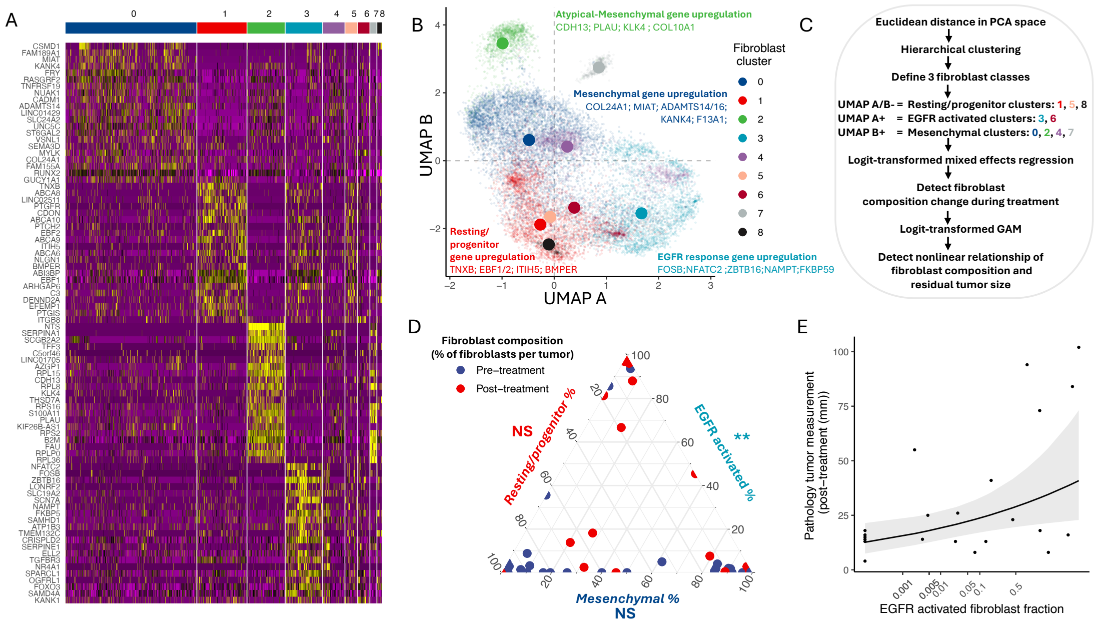

Appendix Figure S4) Unbiased fibroblast clustering reveals distinct fibroblast cell categories, and the frequency of the EGFR activated category increases during treatment and is non-linearly associated to residual tumor size post-treatment. A) Unbiased clustering of fibroblast cells (n= 22916) into 8 subpopulations with distinct gene expression profiles. Cells clustered based on normalized (log(1+CPM)) and scaled (mean=0, sd=1) gene expression of variable genes (n=2000, identified using vst method in Seurat). Linear dimension reduction was performed using PCA and based on the Euclidean distances of cells in this space, a shared nearest neighbor (SNN) graph was constructed, and 9 fibroblast cell clusters (x label color) were identified using the Leiden algorithm. Differential expression analysis was performed to identify genes upregulated in each cluster. Heatmap shows the 20 most upregulated genes (x axis; marker genes) in each of the 4 major cell clusters. Tile coloration indicates scales gene expression (yellow= high; purple=low). **B)** Overlay of fibroblast cluster annotations (color) onto the single cell fibroblast UMAP landscape (axes; small points=single cells) shows strong agreement in inferred fibroblast similarity between approaches. Fibroblast cluster centroids (large points) calculated by the median UMAP dimension scores. Biological interpretation of clusters (bold text) determined by biological function of marker genes (non-bold text). **C)** Workflow to use unbiased clustering results to identify fibroblasts phenotypes enriched post treatment and their association with residual tumor size from pathology. Hierarchical clustering was applied to the Euclidean distance between fibroblast cluster centroids (in PCA space) to group higher resolution clusters into three fibroblast categories: EGFR activated, mesenchymal and resting/progenitor fibroblasts. These fibroblast categories were verified to correspond to cells in distinct quadrants of UMAP space. We next quantified the fraction of fibroblasts within each tumor sample classified into each of the three clustering-based categories. The change in the fraction of fibroblasts in each category pre- to post-treatment was assessed, using logit-transformed mixed effects regression to account for patient specific variation in initial fibroblast composition. The post-treatment (logit-transformed) frequency of each fibroblast category was then used in a generalized additive model (GAM) to predict post-treatment residual tumor size (pathology measured longest length (mm)). **D)** Ternary plot showing the fibroblast composition of tumor samples pre- (blue) and post-treatment (red) in terms of the fraction of fibroblasts classified into the three fibroblast categories (points= fibroblast composition in a specific tumor sample). Linear mixed effects model shows significant increase in the logit-proportion of EGFR activated fibroblasts post-

treatment (est=5.7, se=1.57, df=24.28, z=3.62, p=0.001). No significant change in the logit-proportion of mesenchymal fibroblasts post-treatment (est=-3.74, se=1.95, df=28.8, z=-1.928, p=0.06). Sample size (n): 15908 fibroblasts from 33 patient tumors with pre- and post-treatment fibroblast samples. **E)** Scatterplot showing the relationship between post-treatment EGFR activated fibroblast fraction (logit scale) and the tumor's post-treatment pathological longest length measurement (mm). Tumors with a greater fraction of fibroblasts in an EGFR activated state after treatment (day 180) were significantly larger post treatment (Generalized additive model (GAM) non-linear trend: eff.df=1,F=6.46,p=0.019). Solid line=nonlinear relationship between post-treatment fibroblast fraction and tumor size. Shaded area=95% confidence interval. (+/- 1.96*SE). Sample size= 22 tumors with matched post-treatment measurement of EGFR activated fibroblast fraction and pathological assessment of tumor size.
