# Supplementary figures and images for: Blocking cancer-fibroblast mutualism inhibits proliferation of endocrine therapy resistant breast cancer
Source: Mol Syst Biol. 2025 May 8;21(7):825–55. doi: 10.1038/s44320-025-00104-6 (PMC12222798; doi:10.1038/s44320-025-00104-6)

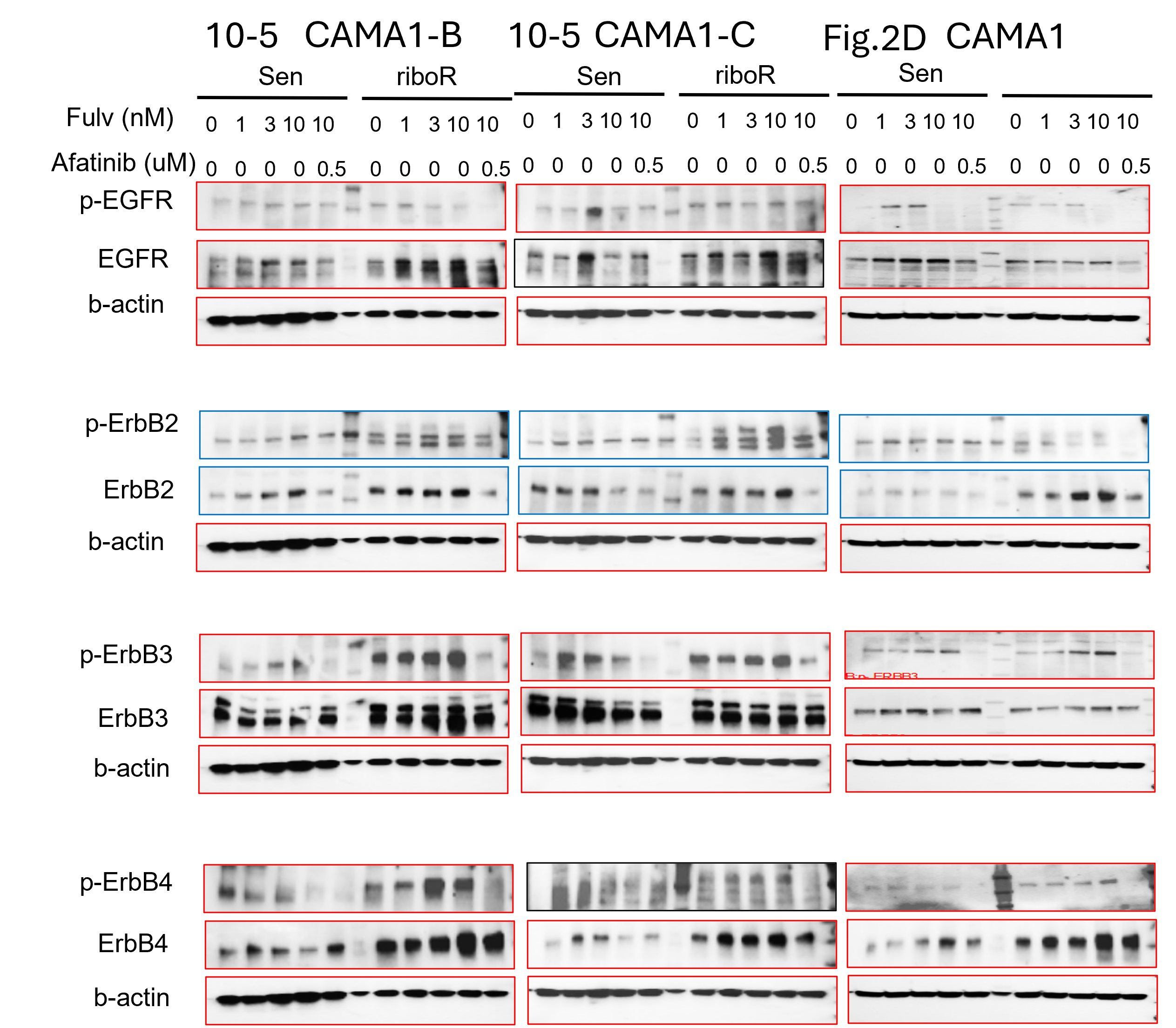

Supplement: Supplementary file 7 — Source data Fig. 2 [file 44320_2025_104_MOESM7_ESM.zip › Figure 2/SourceData_Figure_2C_Images_Western ERBB Fulv and Afatinib/CAMA1 all.tif]

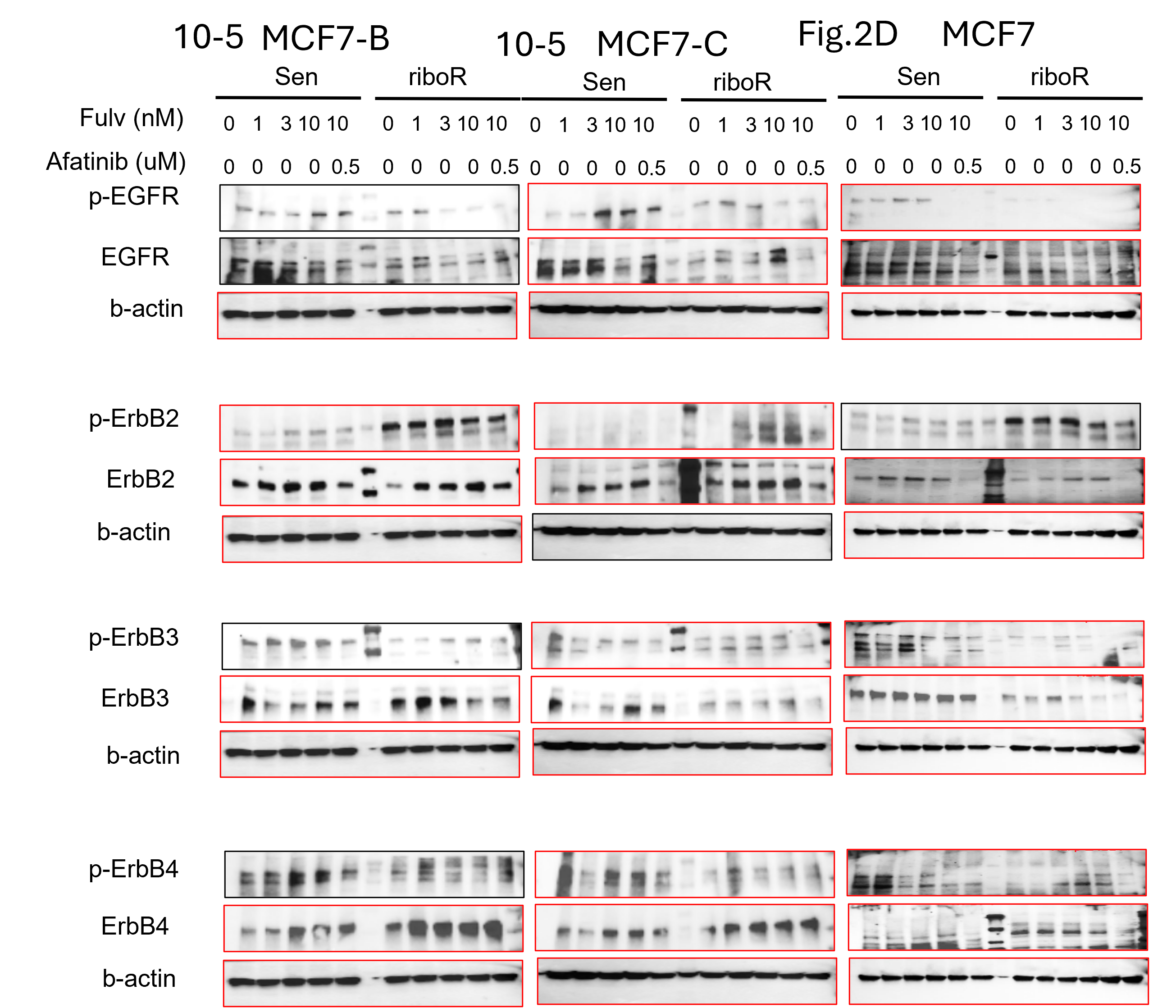

Supplement: Supplementary file 7 — Source data Fig. 2 [file 44320_2025_104_MOESM7_ESM.zip › Figure 2/SourceData_Figure_2C_Images_Western ERBB Fulv and Afatinib/MCF7 all.tif]

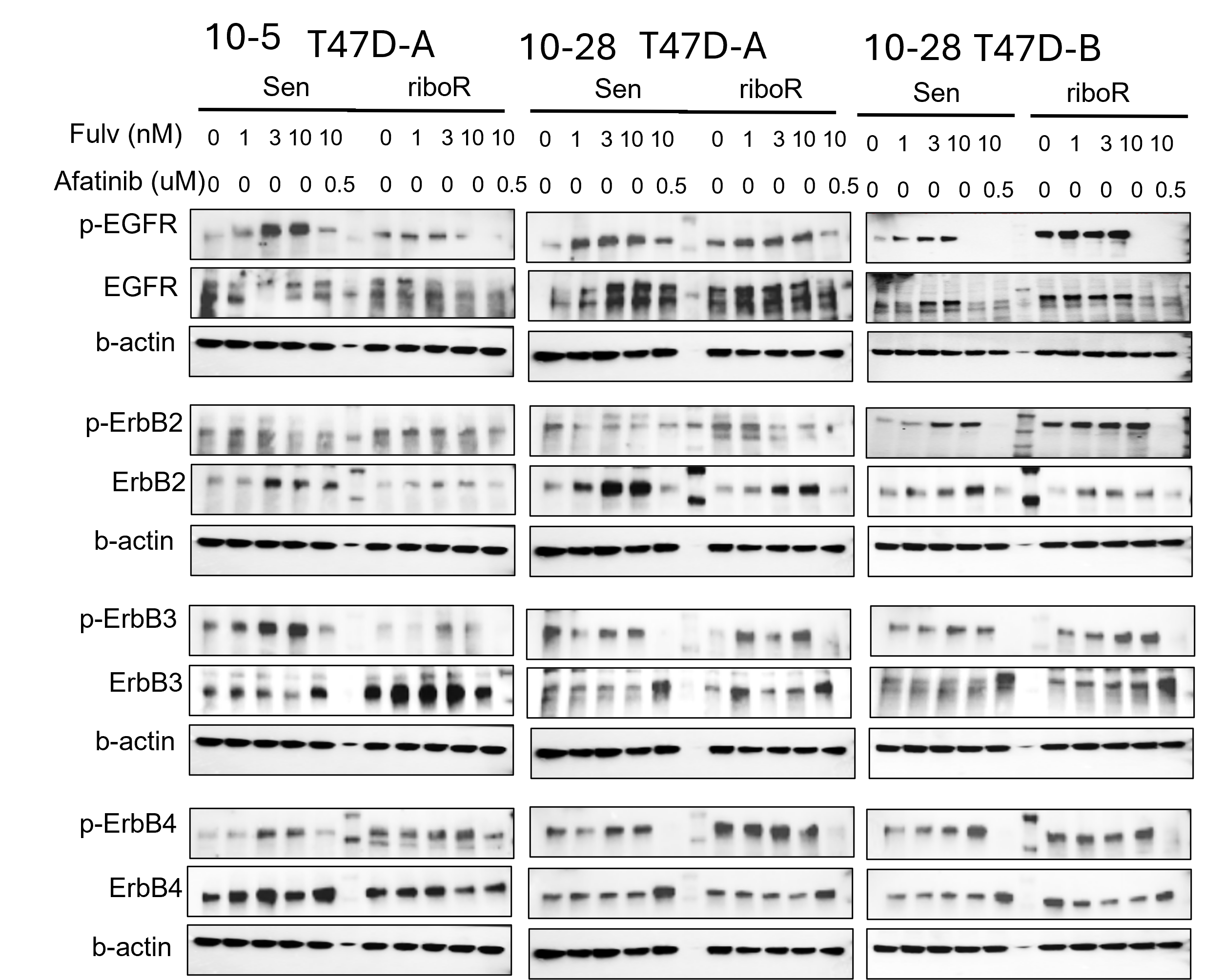

Supplement: Supplementary file 7 — Source data Fig. 2 [file 44320_2025_104_MOESM7_ESM.zip › Figure 2/SourceData_Figure_2C_Images_Western ERBB Fulv and Afatinib/T47D all.tif]

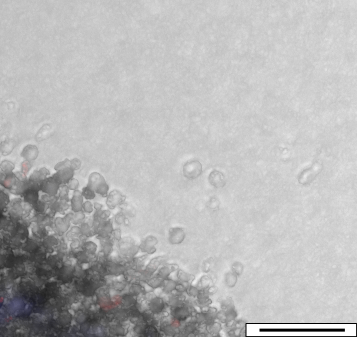

Supplement: Supplementary file 11 — Source data Fig. 6 [file 44320_2025_104_MOESM11_ESM.zip › Figure 6/SourceData_Figure_6C_Plate170_CAMA1RiboRCer2_Well4A_D7-Afatinib_Zoom_J.tif]

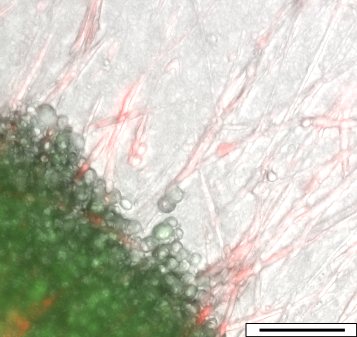

Supplement: Supplementary file 11 — Source data Fig. 6 [file 44320_2025_104_MOESM11_ESM.zip › Figure 6/SourceData_Figure_6C_Plate168_CAMA1SensV2_Well1C_D7-DMSO_Zoom_C.tif]

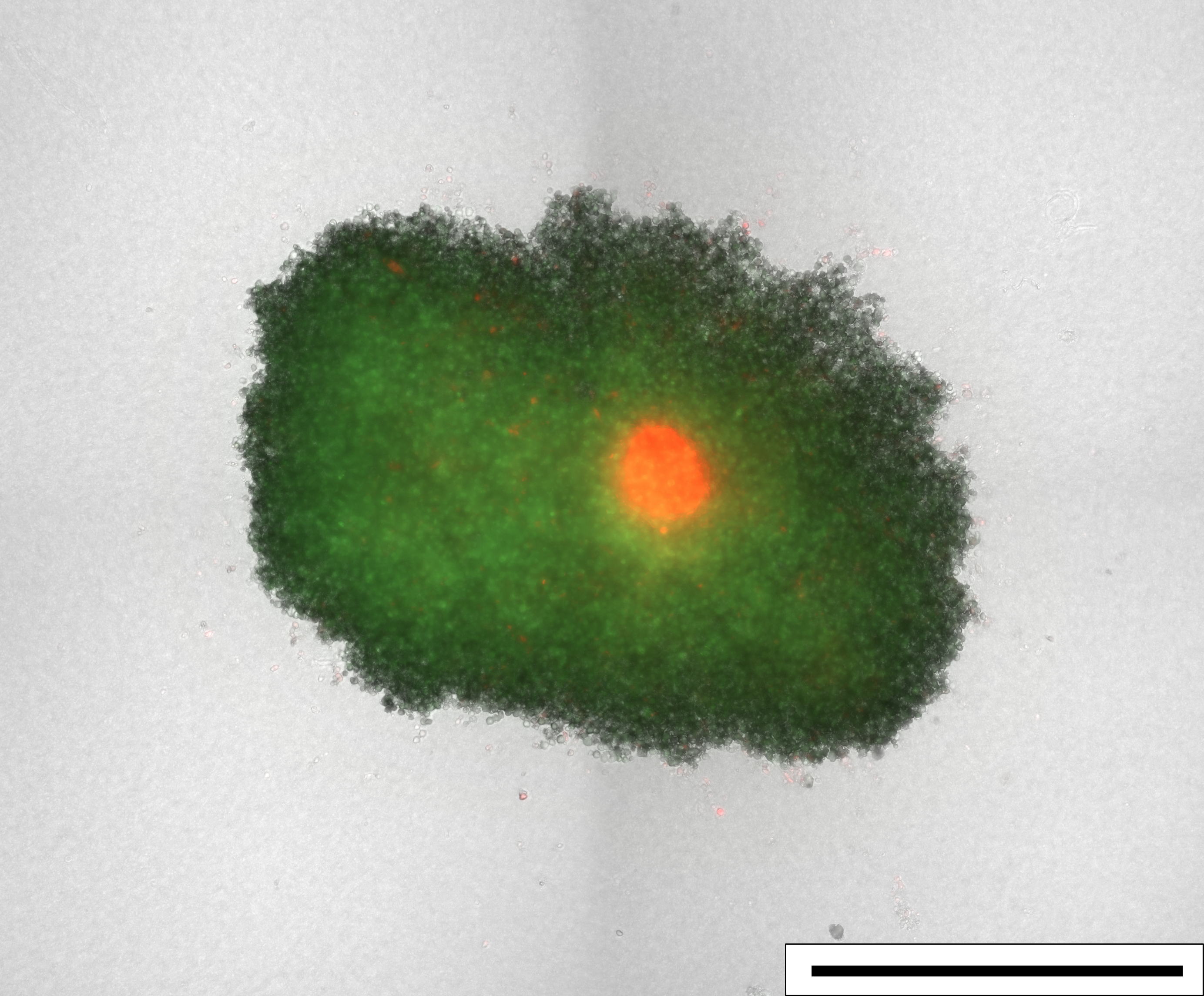

Supplement: Supplementary file 11 — Source data Fig. 6 [file 44320_2025_104_MOESM11_ESM.zip › Figure 6/SourceData_Figure_6C_Plate168_CAMA1SensV2_Well4C_D7-Afatinib_F.tif]

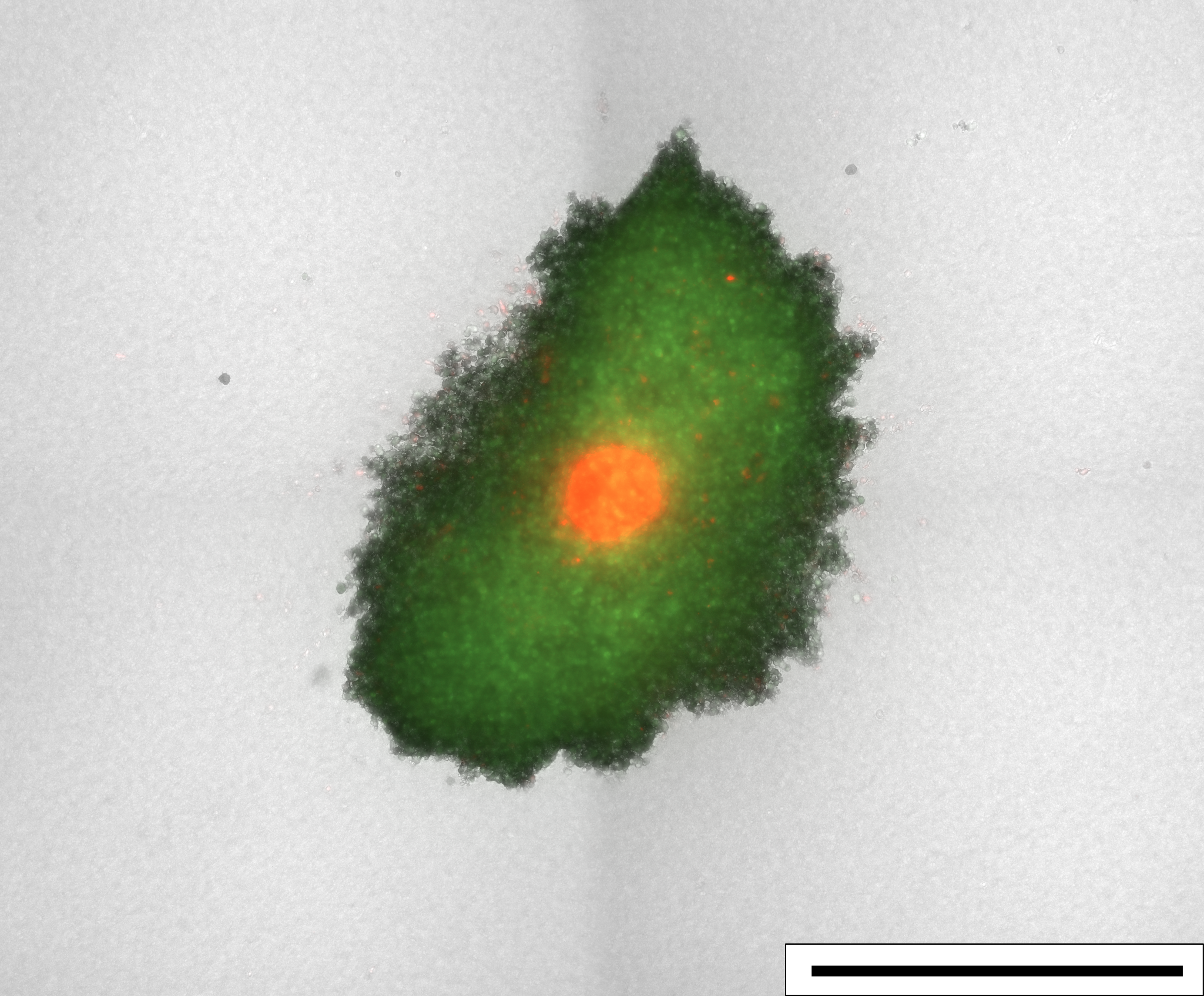

Supplement: Supplementary file 11 — Source data Fig. 6 [file 44320_2025_104_MOESM11_ESM.zip › Figure 6/SourceData_Figure_6C_Plate168_CAMA1SensV2_Well4B_D7-Afatinib_E.tif]

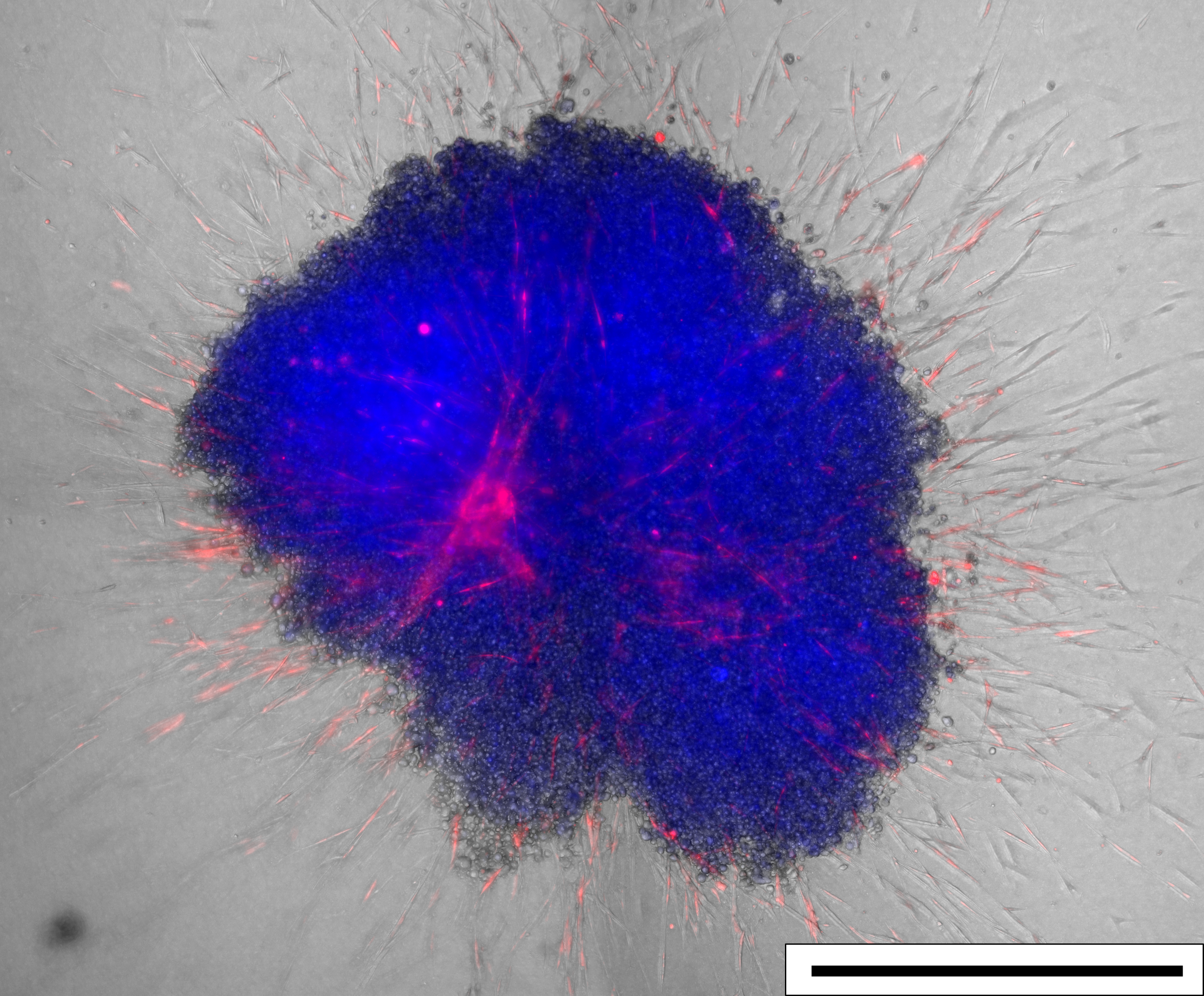

Supplement: Supplementary file 11 — Source data Fig. 6 [file 44320_2025_104_MOESM11_ESM.zip › Figure 6/SourceData_Figure_6C_Plate170_CAMA1RiboRCer2_Well1B_D7-DMSO_H.tif]

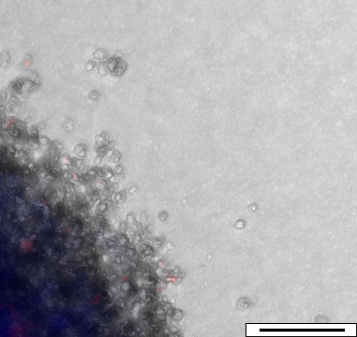

Supplement: Supplementary file 11 — Source data Fig. 6 [file 44320_2025_104_MOESM11_ESM.zip › Figure 6/SourceData_Figure_6C_Plate170_CAMA1RiboRCer2_Well4B_D7-Afatinib_Zoom_K.tif]

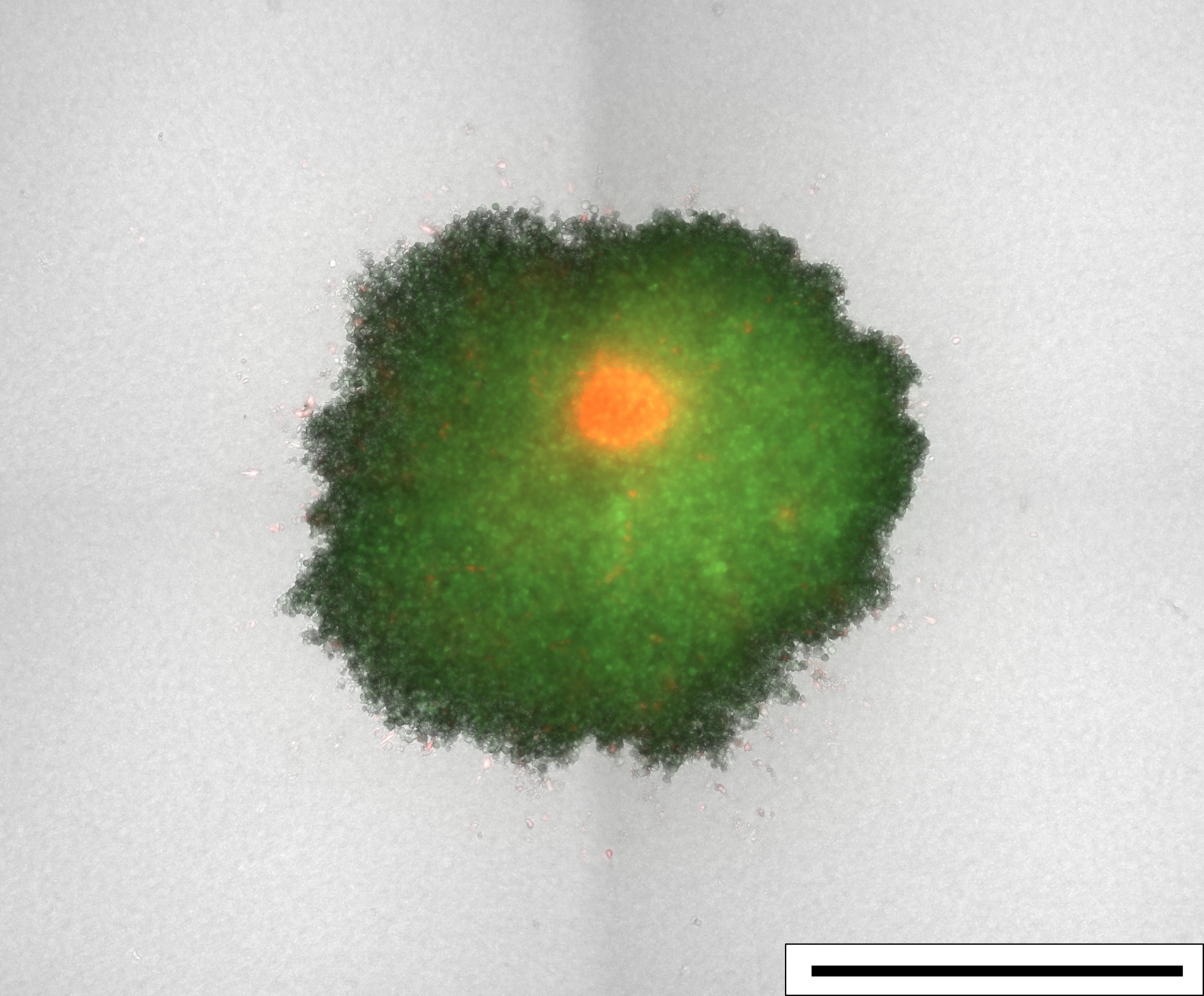

Supplement: Supplementary file 11 — Source data Fig. 6 [file 44320_2025_104_MOESM11_ESM.zip › Figure 6/SourceData_Figure_6C_Plate168_CAMA1SensV2_Well4A_D7-Afatinib_D.tif]

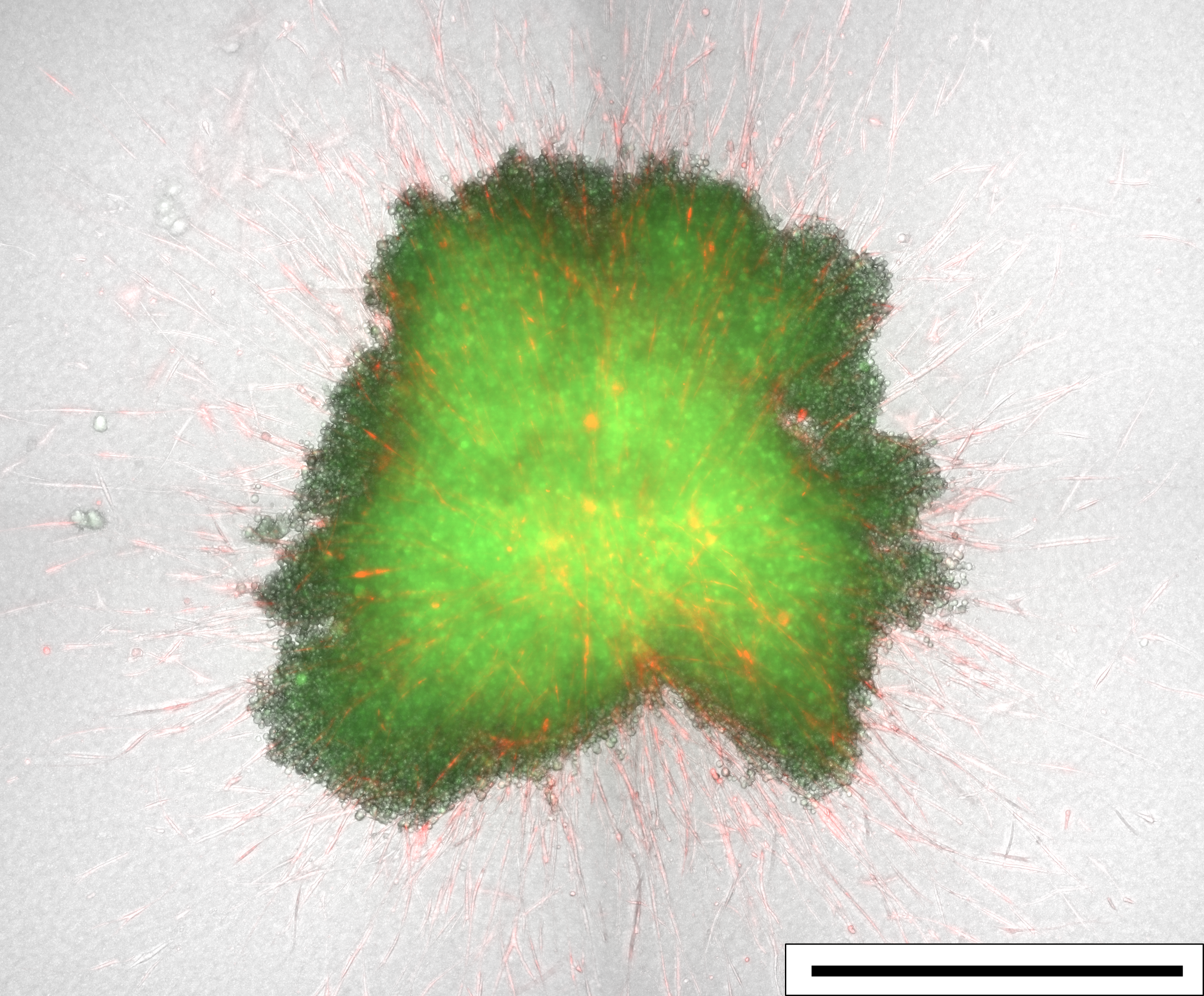

Supplement: Supplementary file 11 — Source data Fig. 6 [file 44320_2025_104_MOESM11_ESM.zip › Figure 6/SourceData_Figure_6C_Plate168_CAMA1SensV2_Well1A_D7-DMSO_A.tif]

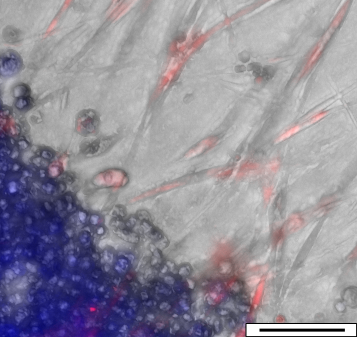

Supplement: Supplementary file 11 — Source data Fig. 6 [file 44320_2025_104_MOESM11_ESM.zip › Figure 6/SourceData_Figure_6C_Plate170_CAMA1RiboRCer2_Well1B_D7-DMSO_Zoom_H.tif]

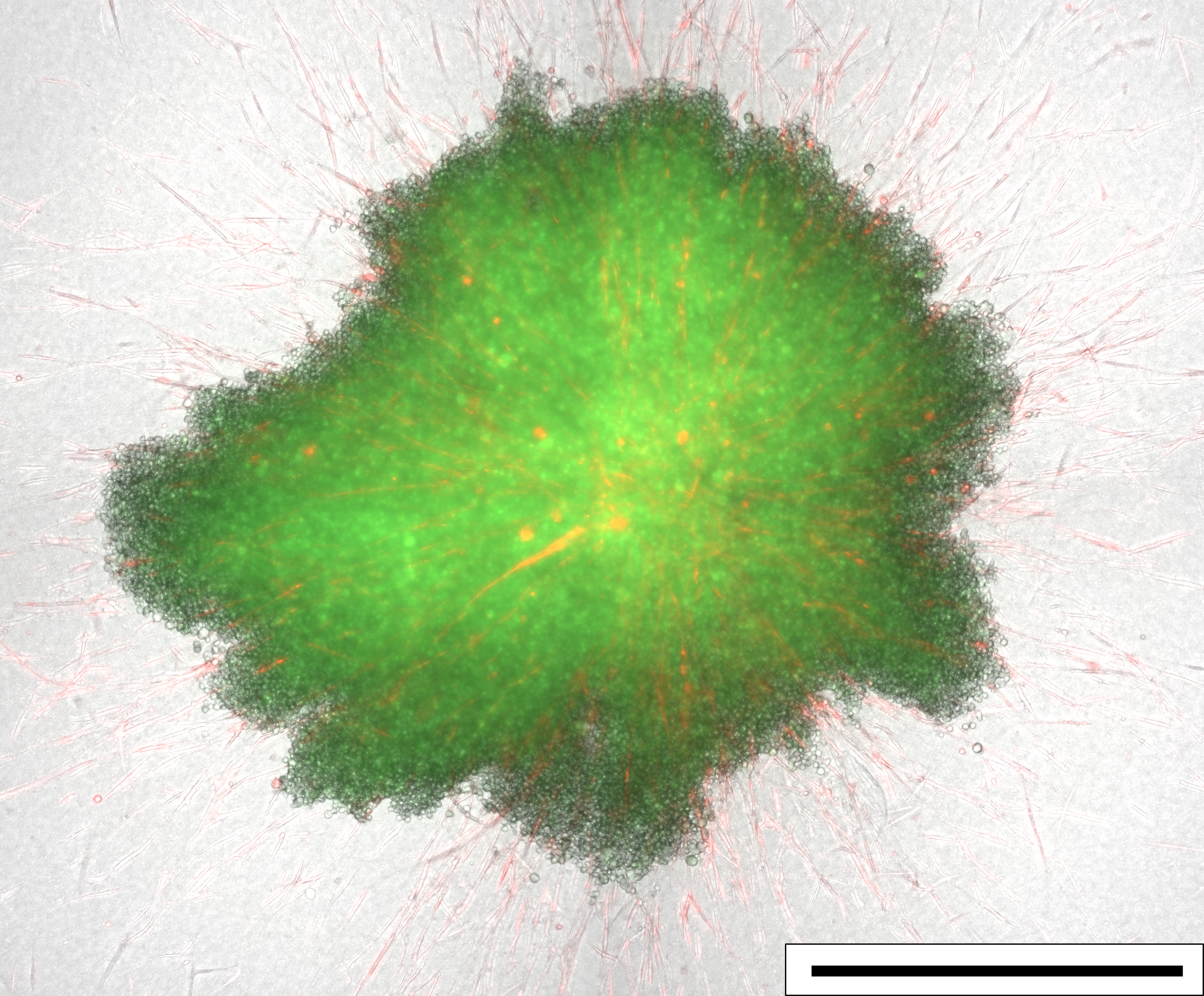

Supplement: Supplementary file 11 — Source data Fig. 6 [file 44320_2025_104_MOESM11_ESM.zip › Figure 6/SourceData_Figure_6C_Plate168_CAMA1SensV2_Well1B_D7-DMSO_B.tif]

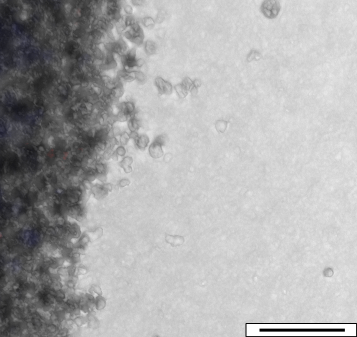

Supplement: Supplementary file 11 — Source data Fig. 6 [file 44320_2025_104_MOESM11_ESM.zip › Figure 6/SourceData_Figure_6C_Plate170_CAMA1RiboRCer2_Well4C_D7-Afatinib_Zoom_L.tif]

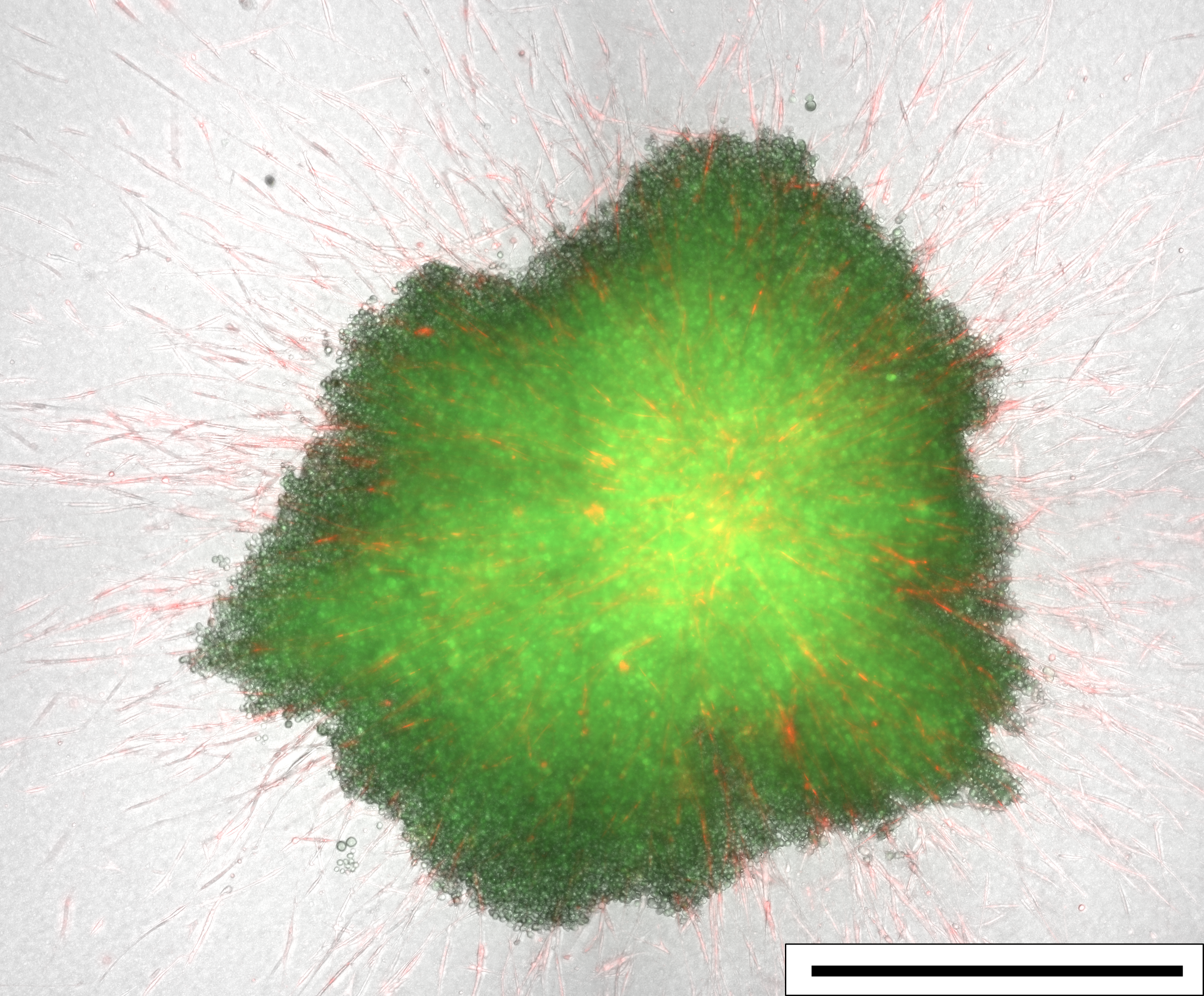

Supplement: Supplementary file 11 — Source data Fig. 6 [file 44320_2025_104_MOESM11_ESM.zip › Figure 6/SourceData_Figure_6C_Plate168_CAMA1SensV2_Well1C_D7-DMSO_C.tif]

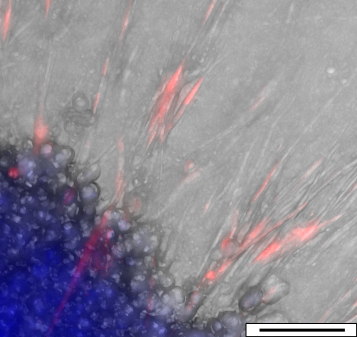

Supplement: Supplementary file 11 — Source data Fig. 6 [file 44320_2025_104_MOESM11_ESM.zip › Figure 6/SourceData_Figure_6C_Plate170_CAMA1RiboRCer2_Well1A_D7-DMSO_Zoom_G.tif]

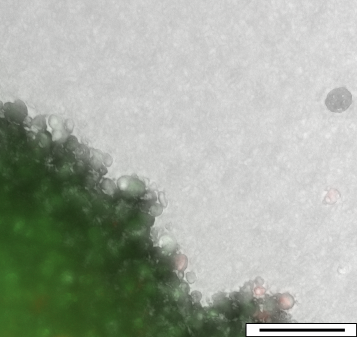

Supplement: Supplementary file 11 — Source data Fig. 6 [file 44320_2025_104_MOESM11_ESM.zip › Figure 6/SourceData_Figure_6C_Plate168_CAMA1SensV2_Well4B_D7-Afatinib_Zoom_E.tif]

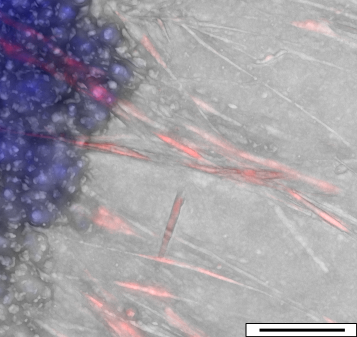

Supplement: Supplementary file 11 — Source data Fig. 6 [file 44320_2025_104_MOESM11_ESM.zip › Figure 6/SourceData_Figure_6C_Plate170_CAMA1RiboRCer2_Well1C_D7-DMSO_Zoom_I.tif]

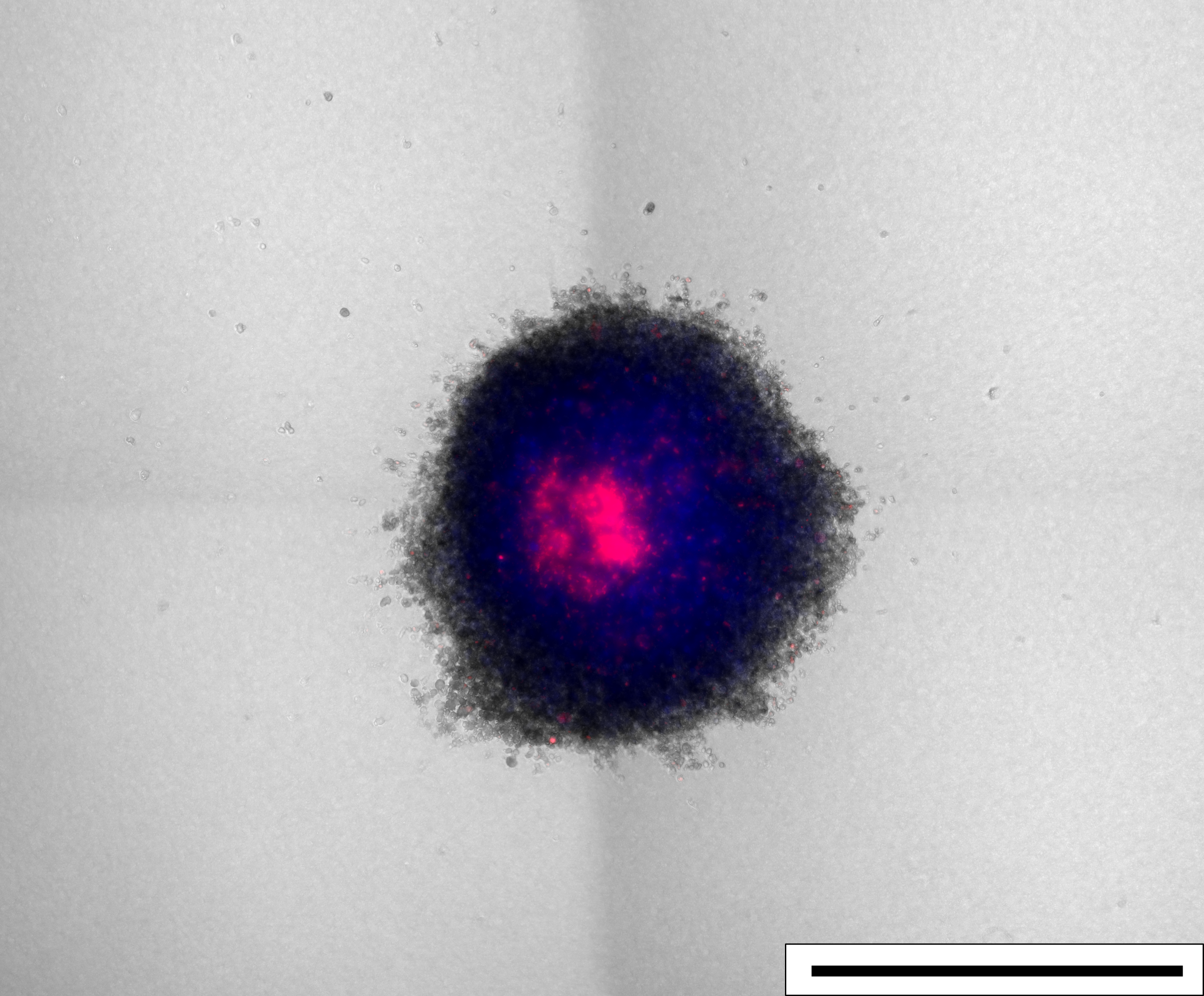

Supplement: Supplementary file 11 — Source data Fig. 6 [file 44320_2025_104_MOESM11_ESM.zip › Figure 6/SourceData_Figure_6C_Plate170_CAMA1RiboRCer2_Well4B_D7-Afatinib_K.tif]

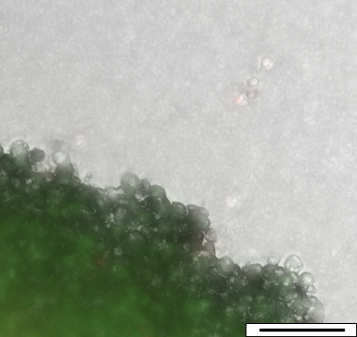

Supplement: Supplementary file 11 — Source data Fig. 6 [file 44320_2025_104_MOESM11_ESM.zip › Figure 6/SourceData_Figure_6C_Plate168_CAMA1SensV2_Well4A_D7-Afatinib_Zoom_D.tif]

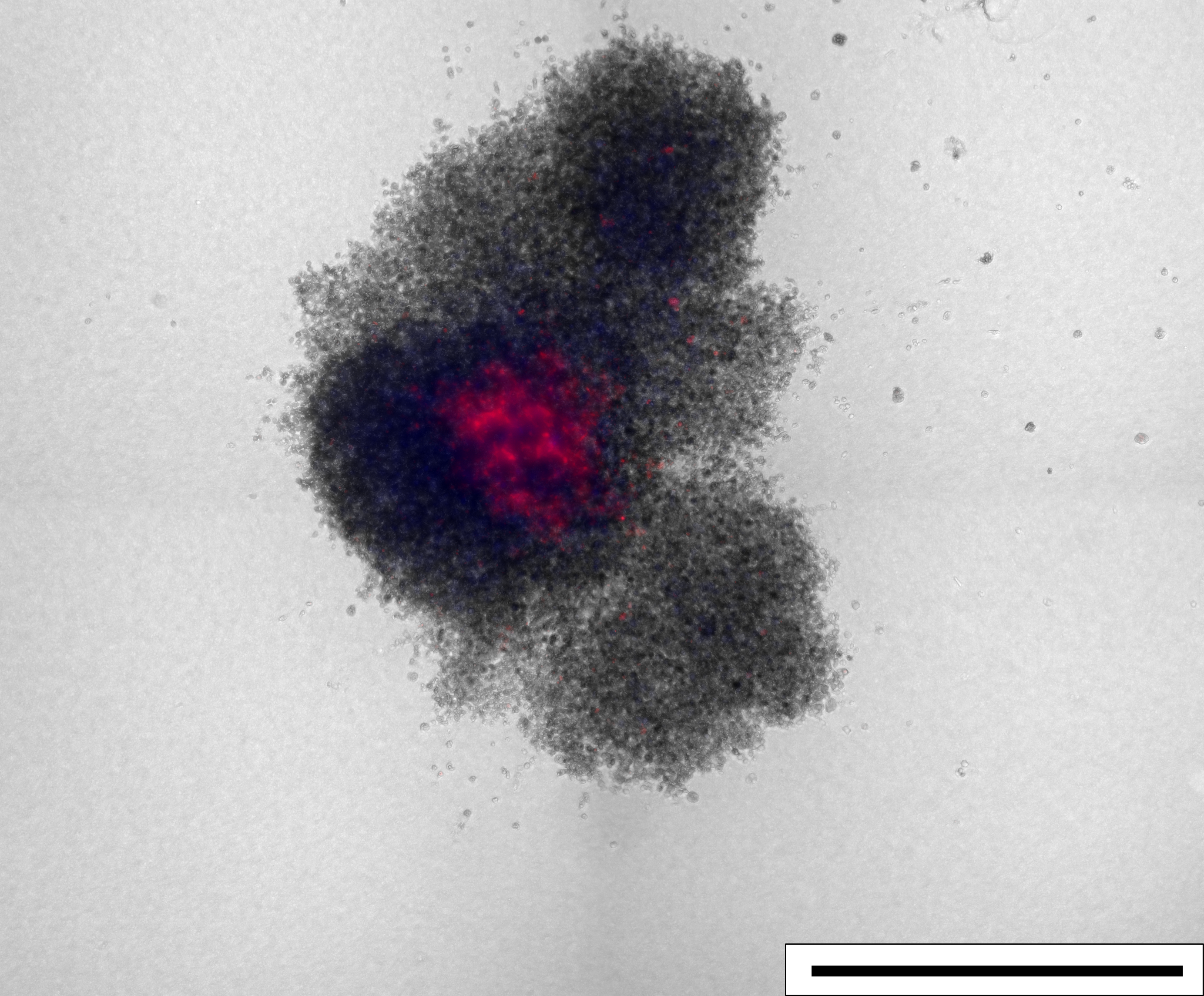

Supplement: Supplementary file 11 — Source data Fig. 6 [file 44320_2025_104_MOESM11_ESM.zip › Figure 6/SourceData_Figure_6C_Plate170_CAMA1RiboRCer2_Well4C_D7-Afatinib_L.tif]

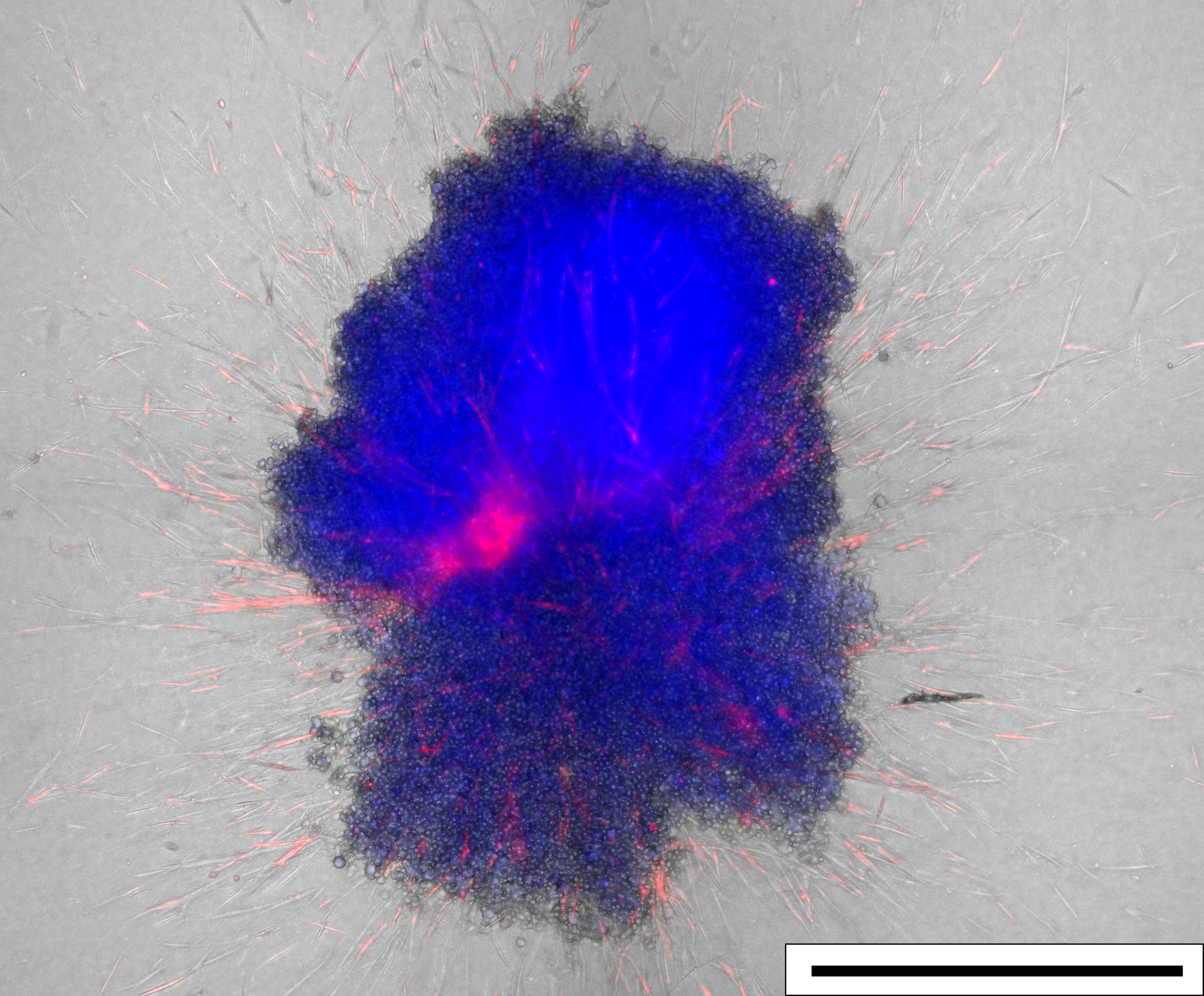

Supplement: Supplementary file 11 — Source data Fig. 6 [file 44320_2025_104_MOESM11_ESM.zip › Figure 6/SourceData_Figure_6C_Plate170_CAMA1RiboRCer2_Well1C_D7-DMSO_I.tif]

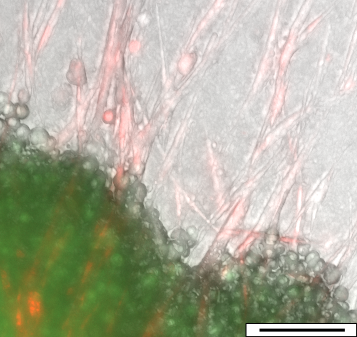

Supplement: Supplementary file 11 — Source data Fig. 6 [file 44320_2025_104_MOESM11_ESM.zip › Figure 6/SourceData_Figure_6C_Plate168_CAMA1SensV2_Well1A_D7-DMSO_Zoom_A.tif]

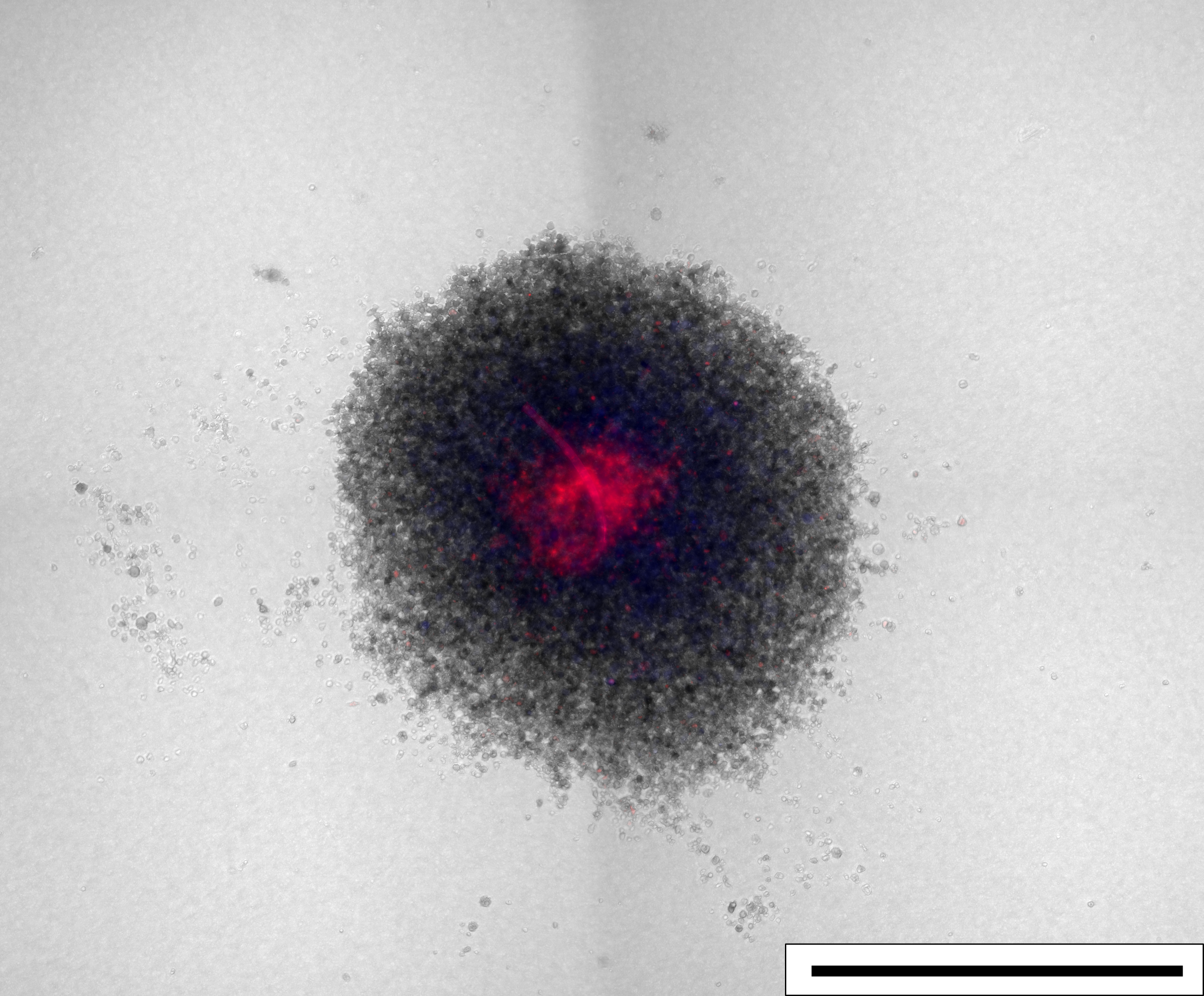

Supplement: Supplementary file 11 — Source data Fig. 6 [file 44320_2025_104_MOESM11_ESM.zip › Figure 6/SourceData_Figure_6C_Plate170_CAMA1RiboRCer2_Well4A_D7-Afatinib_J.tif]

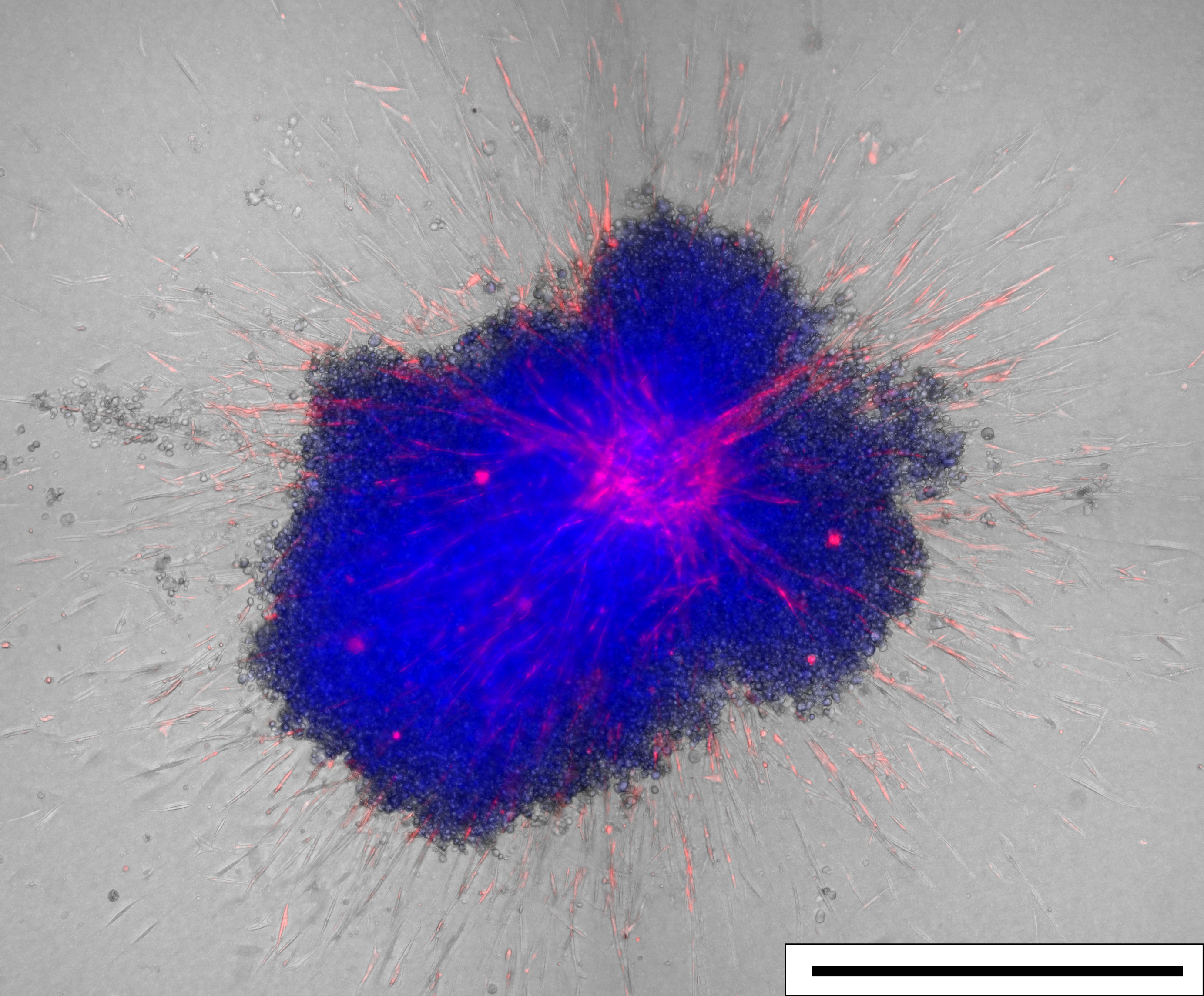

Supplement: Supplementary file 11 — Source data Fig. 6 [file 44320_2025_104_MOESM11_ESM.zip › Figure 6/SourceData_Figure_6C_Plate170_CAMA1RiboRCer2_Well1A_D7-DMSO_G.tif]

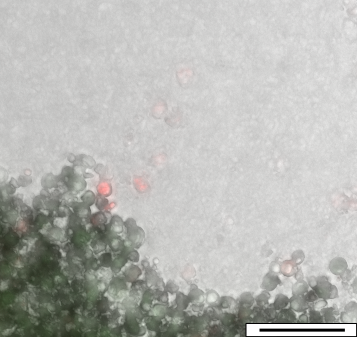

Supplement: Supplementary file 11 — Source data Fig. 6 [file 44320_2025_104_MOESM11_ESM.zip › Figure 6/SourceData_Figure_6C_Plate168_CAMA1SensV2_Well4C_D7-Afatinib_Zoom_F.tif]

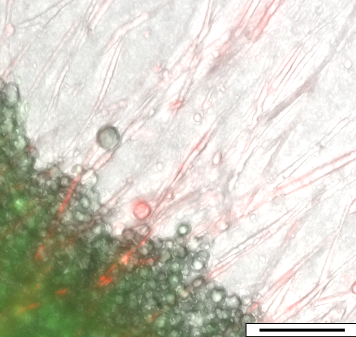

Supplement: Supplementary file 11 — Source data Fig. 6 [file 44320_2025_104_MOESM11_ESM.zip › Figure 6/SourceData_Figure_6C_Plate168_CAMA1SensV2_Well1B_D7-DMSO_Zoom_B.tif]
