# Supplementary material for: Blocking cancer-fibroblast mutualism inhibits proliferation of endocrine therapy resistant breast cancer
Source: Mol Syst Biol. 2025 May 8;21(7):825–55. doi: 10.1038/s44320-025-00104-6 (PMC12222798; doi:10.1038/s44320-025-00104-6)
Supplement: Supplementary file 12 — Expanded View Figures [file 44320_2025_104_MOESM12_ESM.pdf]

Expanded View Figures

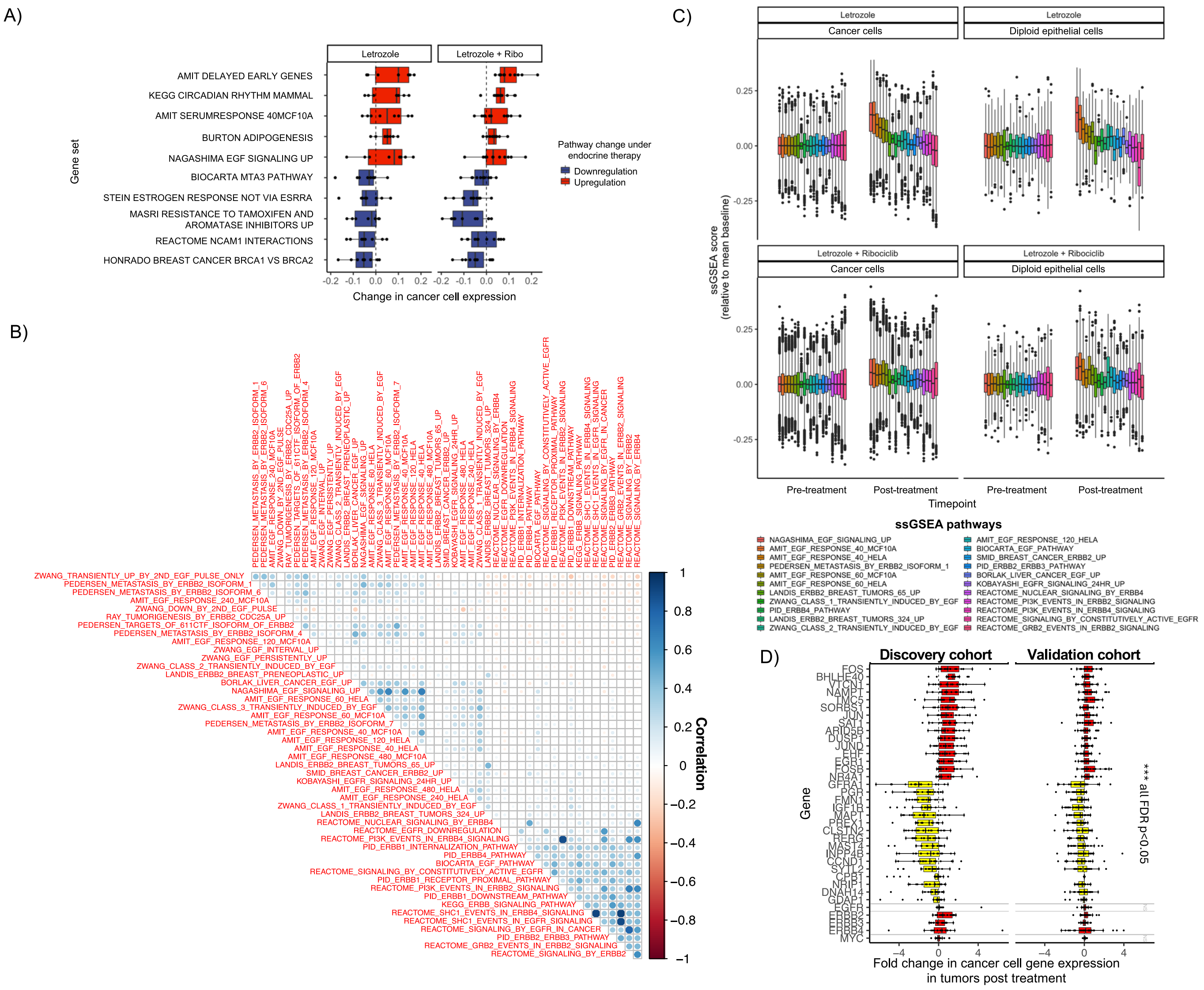

Figure EV1. ERBB pathway upregulation during treatment in endocrine +/- CDK4/6i-resistant cancer cells of patient tumors.

(**A**) Cancer phenotypic changes during endocrine and combination ribociclib treatment. Summary of the top 5 ssGSEA pathways upregulated (red) and downregulated (blue) following endocrine therapy (ET) in patients treated with letrozole alone (left) and combination ribociclib (ribo) treatment (right). Points show the difference in mean ssGSEA pathway scores of cancer cells in a tumor pre- versus post treatment (positive values = increase activity within tumor post treatment). Box elements show median (center line), upper and lower quartiles (hinges) and 1.5*interquartile range (whiskers=minima/maxima) of changes in pathway activity across patient tumors. (**B**) Positive correlation among ERBB pathway scores across cancer cells. Correlation plot among 47 ssGSEA pathways (axes labels) contributing to the overall ERBB activation score. Coloration indicates the strength of correlation between pairs of pathways (blue=high, red=low correlation). Point sizes proportional to the absolute value of the correlation coefficient. (**C**) ERBB pathway activation in cancer and diploid epithelial cells during treatment. Boxplots showing post-treatment ERBB pathway activation of cancer (left) and diploid epithelial cells (right) resistant to letrozole alone (top) or combination ribociclib treatment (bottom). Points show ERBB pathway scores (*y* axis) of single cells as measured by 22 different ssGSEA scores (colors), all showing overall significant increases in post-treatment resistant cancer cells. The extent of pathway activation in diploid epithelial cells was consistent with that observed in cancer cells. Box elements show median (center line), upper and lower quartiles (hinges) and 1.5*interquartile range (whiskers=minima/maxima) of changes in pathway activity across patient tumors. (**D**) Genes modulated in resistant patient cancer cells. Boxplot of the top 15 most consistently upregulated and downregulated genes (red versus yellow) in cancer cells following treatment. Across patient tumors, cancer cells resistant to treatment (sampled post treatment) showed consistent upregulation (red) of transcription factors downstream of ERBB signaling (e.g., FOS, FOSB, JUN and JUND) and downregulation (yellow) of genes involved in estrogen-dependent growth (e.g., PGR and MAPT). At the bottom, we present the changes in the four ERRB receptor genes and MYC, a key downstream transcription factor. Points indicate the fold change in the average gene expression of cancer cells of a tumor post treatment compared to pre-treatment (positive values= increased expression during treatment). Box elements represent median (center line), upper/lower quartiles (hinges), 1.5*interquartile range (whiskers=minima/maxima) of gene expression changes across tumors from the discovery cohort. All FDR-corrected ANOVA *P* values less than 0.05. Gene specific statistics (including exact *P* values) are provided in Table EV3. Sample sizes in (**A–D**): Discovery:: 57,403 tumor-derived cancer cells (36,825 pre-treatment, 20,578 post treatment) from 16 patients with paired pre- and post-treatment samples with >20 cancer cells ($n = 32$ samples), Validation:: 163245 tumor-derived single cells (51,088 pre-treatment, 25,727 post treatment) from 25 patients with paired pre- and post-treatment samples with >20 cancer cells ($n = 50$ samples).

© The Author(s)

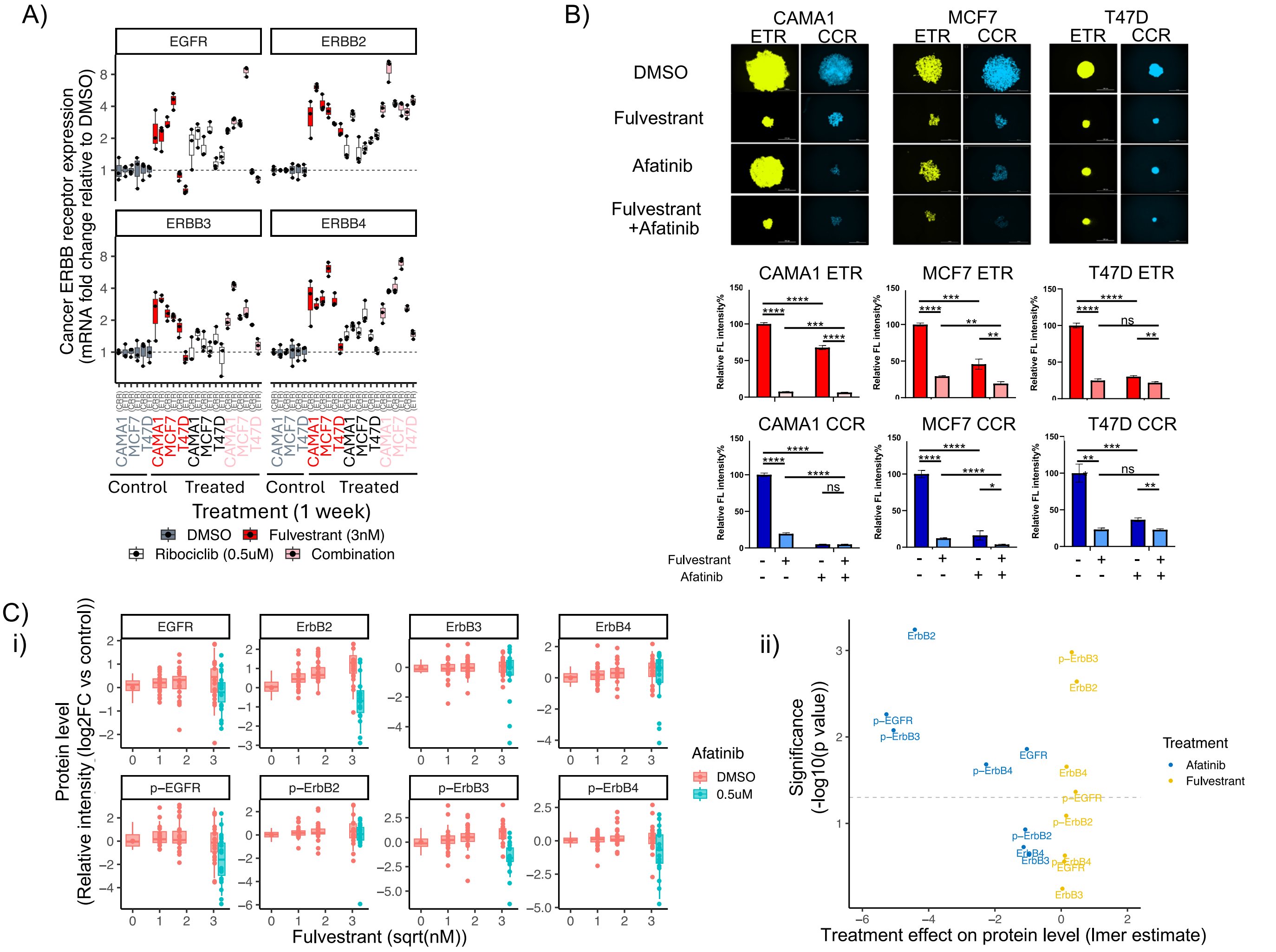
A)
EGFR
ERBB2
ERBB3
ERBB4
Cancer ERBB receptor expression
(mRNA fold change relative to DMSO)
Control Treated Control Treated
Treatment (1 week)
DMSO Fulvestrant (3nM)
Ribociclib (0.5uM) Combination
B)
CAMA1 MCF7 T47D
ETR CCR ETR CCR ETR CCR
DMSO
Fulvestrant
Afatinib
Fulvestrant +Afatinib
CAMA1 ETR
MCF7 ETR
T47D ETR
CAMA1 CCR
MCF7 CCR
T47D CCR
Relative FL intensity%
Fulvestrant
Afatinib
C)
i)
EGFR
ErbB2
ErbB3
ErbB4
p−EGFR
p−ErbB2
p−ErbB3
p−ErbB4
Protein level
(Relative intensity (log2FC vs control))
Fulvestrant (sqrt(nM))
Afatinib
DMSO
0.5uM
ii)
Significance
(-log10(p value))
Treatment effect on protein level (lmer estimate)
Treatment
Afatinib
Fulvestrant

◀ **Figure EV2. In vitro validation of ERBB pathway activation during endocrine or CDK4/6i treatment and the control of cancer cell growth by targeted therapy inhibiting the ERBB pathway.**

(**A**) Validation in vitro that ER+ breast cancer cells broadly upregulate ERBB receptor gene expression under endocrine and CDK4/6 inhibition treatment. Fold change of ERBB growth factor receptor mRNA expression (panels), measured by qPCR in three paired cancer cell lines (*x* axis: CAMA-1, MCF-7 and T47D endocrine therapy-resistant (ETR) and combination ribociclib-resistant (CRR)) under endocrine therapy (fulvestrant 3 nM), CDK4/6 inhibition (ribociclib 0.5 μM) or combination treatments (fulvestrant 3 nM plus ribociclib 0.5 μM) compared to DMSO control. Endocrine and endocrine+CDK4/6i combination treatment, but not CDK4/6i alone, broadly increased ERBB growth factor receptor expression after 7 days of treatment compared to DMSO control (linear mixed model expression change under:: Endocrine:Est=1.83, se=0.19, df=276, $t = 9.77$, $P = 2e-16$, CDK4/6i:Est=0.64, se= 0.19, df=276, $t = 3.41$, $P = 7.4e-4$, Combination: Est=2.43, se=0.19, df=276, $t = 12.96$, $P = 2e-16$; Sample size=288 qPCR samples, 6 cell lines, 3 replicates per gene ($n = 4$) and treatment ($n = 4$)). Box elements represent median (center line), upper/lower quartiles (hinges), 1.5*interquartile range (whiskers=minima/maxima) of ERBB receptor fold change versus control. (**B**) Combination of fulvestrant and afatinib inhibits spheroid growth of 3D cancer monocultures. (**A**) Representative images of spheroid growth of sensitive (Venus, green) or resistant (CFP, blue) cells cultured in fulvestrant (5 nM), afatinib (1.25 μM for CAMA-1, 1.5 μM for MCF-7, and 2.5 μM for T47D), or combination treated media for 18 days. Bars=1000 μm. (**B**) Fluorescence intensity of sensitive and resistant cells under various treatment conditions. Data are represented as average of three replicates ± standard deviation (SD). *$P < 0.05$, **$P < 0.01$, ***$P < 0.001$, ****$P < 0.0001$. T test: differential intensity between treatments:: CAMA-1 ETR: Fulvestrant vs. DMSO $P = 0.0001$, Afatinib vs.DMSO $P = 0.0001$, Fulvestrant+Afatinib vs. Afatinib $P = 0.0001$, Fulvestrant+Afatinib vs. Fulvestrant $P = 0.0006$; MCF-7 ETR: Fulvestrant vs. DMSO $P = 0.0001$, Afatinib vs. DMSO $P = 0.0002$, Fulvestrant+Afatinib vs. Afatinib $P = 0.0036$, Fulvestrant+Afatinib vs.Fulvestrant $P = 0.0029$; T47D ETR: Fulvestrant vs.DMSO $P = 0.0001$, Afatinib vs.DMSO $P = 0.0001$, Fulvestrant+Afatinib vs. Afatinib $P = 0.0019$, Fulvestrant+Afatinib vs. Fulvestrant $P = 0.1246$; CAMA-1 CCR: Fulvestrant vs.DMSO $P = 0.0001$, Afatinib vs.DMSO $P = 0.0001$, Fulvestrant+Afatinib vs. Afatinib $P = 0.346$, Fulvestrant+Afatinib vs. Fulvestrant $P = 0.0001$; MCF-7 CCR: Fulvestrant vs. DMSO $P = 0.0001$, Afatinib vs.DMSO $P = 0.0001$, Fulvestrant+Afatinib vs. Afatinib $P = 0.0265$, Fulvestrant+Afatinib vs. Fulvestrant $P = 0.0001$; T47D CCR: Fulvestrant vs.DMSO $P = 0.0004$, Afatinib vs.DMSO $P = 0.0009$, Fulvestrant+Afatinib vs. Afatinib $P = 0.0013$, Fulvestrant+Afatinib vs. Fulvestrant $P = 0.808$. (**C**) Western blot quantification shows ERBB receptor protein levels increased under fulvestrant treatment and reduced under afatinib across cancer cell lines and experimental replicates. Western blot intensities were measured using ImageJ. Relative intensities of each protein were determined by first normalizing to B-actin and calculating log2 fold changes compared to DMSO control within each cell line. Panel i shows the positive effect of fulvestrant (x axis) and the inhibitory effect of afatinib (color) on the levels of total (top row) and phosphorylated (bottom row) ERBB receptor protein across family members (columns). Points indicate measured relative intensities (quantified western blot bands). Linear mixed-effects regressions (lmer) were fitted for each protein, using data from across treatments, cell lines and experimental replicates to quantify the fulvestrant and afatinib treatment effect sizes, whilst accounting for the cell line and replicate-specific variation in Western intensities, both in the DMSO control and the treatment effects. Boxplots indicate the range of fitted model estimates across replicates within each treatment group (central line=median prediction; lower/upper hinges correspond to first/third quartiles; lower/upper whisker (minima/maxima) extends from the hinge to the largest value less than 1.5 * IQR from the lower/upper hinge). The goodness of fit to the data supports significance estimates of treatment effects. Panel ii shows the lmer (Linear mixed-effects regression) estimated impacts of fulvestrant treatment (yellow) and afatinib treatment (blue) on ERBB protein levels (x axis) and accompanying p values for each ERBB protein (y axis). Across proteins, fulvestrant treatment consistently increased protein levels. Conversely, afatinib consistently reduced them. Sample size = 720 Western blots: 6 cell lines, 8 proteins, 5 treatments, 3 experimental replicates.

 © The Author(s)

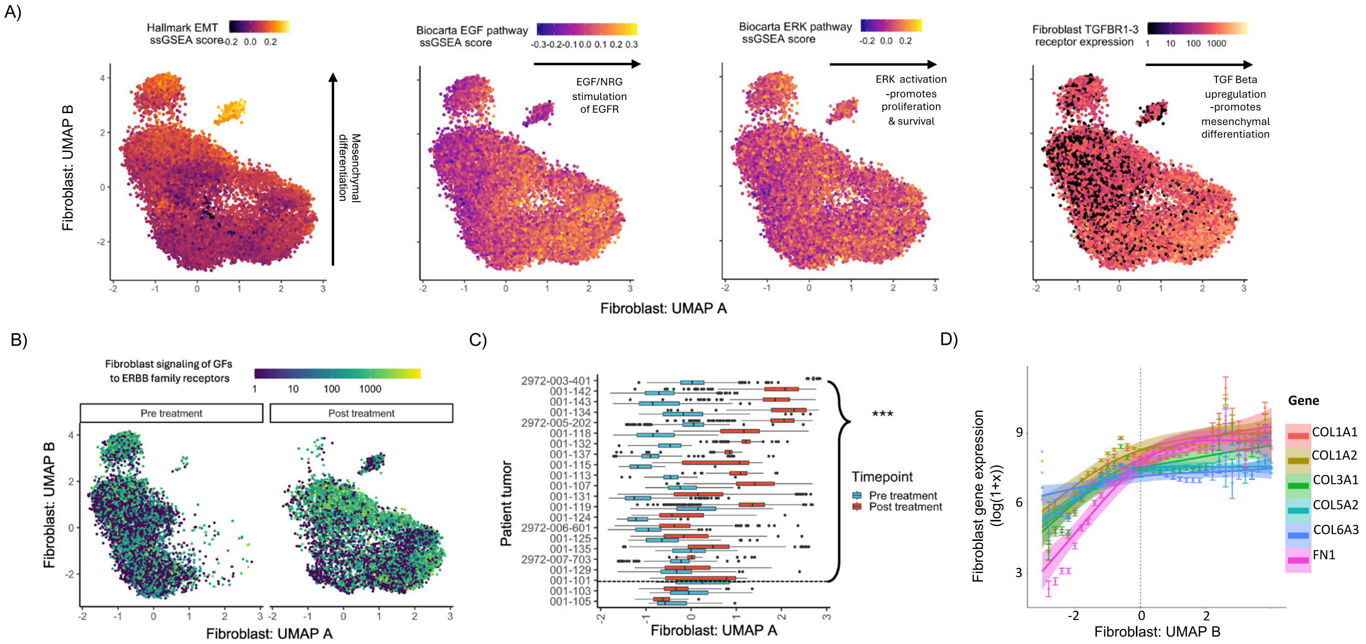

Figure EV3. Single-cell phenotypic heterogeneity and ERBB growth factor signaling of fibroblasts of endocrine +/- CDK4/6i treated patient tumors.

(**A**) Fibroblast differentiation across the phenotype landscape described by UMAP projection. Points represent single-cell phenotypes. Cells with similar phenotypes are positioned more closely together. The major axes of phenotypic variation correspond to fibroblast EGFR-activated proliferation (UMAP A: *x* axis: high Biocarta EGF and ERK pathway activation) and mesenchymal fibroblast differentiation (UMAP B: *y* axis: high hallmark EMT pathway activation). The biological interpretation of the UMAP dimensions (axis labels) were determined by assessment of the genes, gene sets and communications that dynamically changed (nonlinearly) along each dimension. Biological interpretation of fibroblast phenotypes supported by unsupervised clustering and differential expression analysis (Appendix Figs. S3 and S4). EGFR-activated fibroblasts showed upregulation of TGFβ receptors that drive SMAD signaling and further mesenchymal differentiation indicating that EGFR activation supports mesenchymal fibroblast differentiation (right panel Color= total single-cell expression across TGFβ receptors 1–3). (**B**) Fibroblast ERBB ligand growth factor signaling activated during treatment in mesenchymal fibroblast cells and a subpopulation of EGFR-activated fibroblasts (color= total single-cell expression of ERBB ligands: NGR1-3, EGF, HBEGF, AREG, TGFA, EMA4D, HLA-A, EFNB1, ICAM1, GNAI2, CDH1, AREG, ANXA1 and ADAM17. (**C**) Fibroblast EGFR-activated proliferation increased consistently across tumors during endocrine treatment (19/22 letrozole treated patient tumors in the discovery cohort) (Linear mixed effect:: Est=1.23, se=0.19, df=21.00, $t = 6.37$, $P = 2.6e\text{-}6$; asterisk signifies significance). Box elements represent median (center line), upper/lower quartiles (hinges), 1.5*interquartile range (whiskers=minima/maxima) of fibroblast EGFR activation proliferation (measured by fibroblast UMAP A) within a tumor pre or post treatment. Similarly, the fraction of fibroblasts classified (using unsupervised clustering) as EGFR-activated cells increased during treatment. Patients with a greater fraction of EGFR-activated fibroblasts post treatment exhibited greater residual tumor size at day 180 (pathological measurement of longest length; Appendix Fig. S3). (**D**) Mesenchymal fibroblast differentiation is associated with increased expression of fibronectin and various collagens which drive tumor fibrosis and cancer cell proliferation through extracellular matrix modification. Fibroblasts were finely classified by differentiation state across the phenotypic landscape (mesenchymal fibroblast differentiation: UMAP B) and the mean (points) and standard error (error bars) of collagen and fibronectin gene expression (color) is shown for each level of differentiation. The distribution of fibroblast differentiation states was discretized into 36 equal sized classes to provide an average of 329 cells per level of differentiation. Generalized additive models (smooth curves with uncertainty regions shaded) characterize the trend in gene expression during differentiation. Sample size in (**A–D**): $n = 22{,}916$ fibroblast cells of the discovery cohort from 22 patient tumors at two paired timepoints (pre/post treatment).

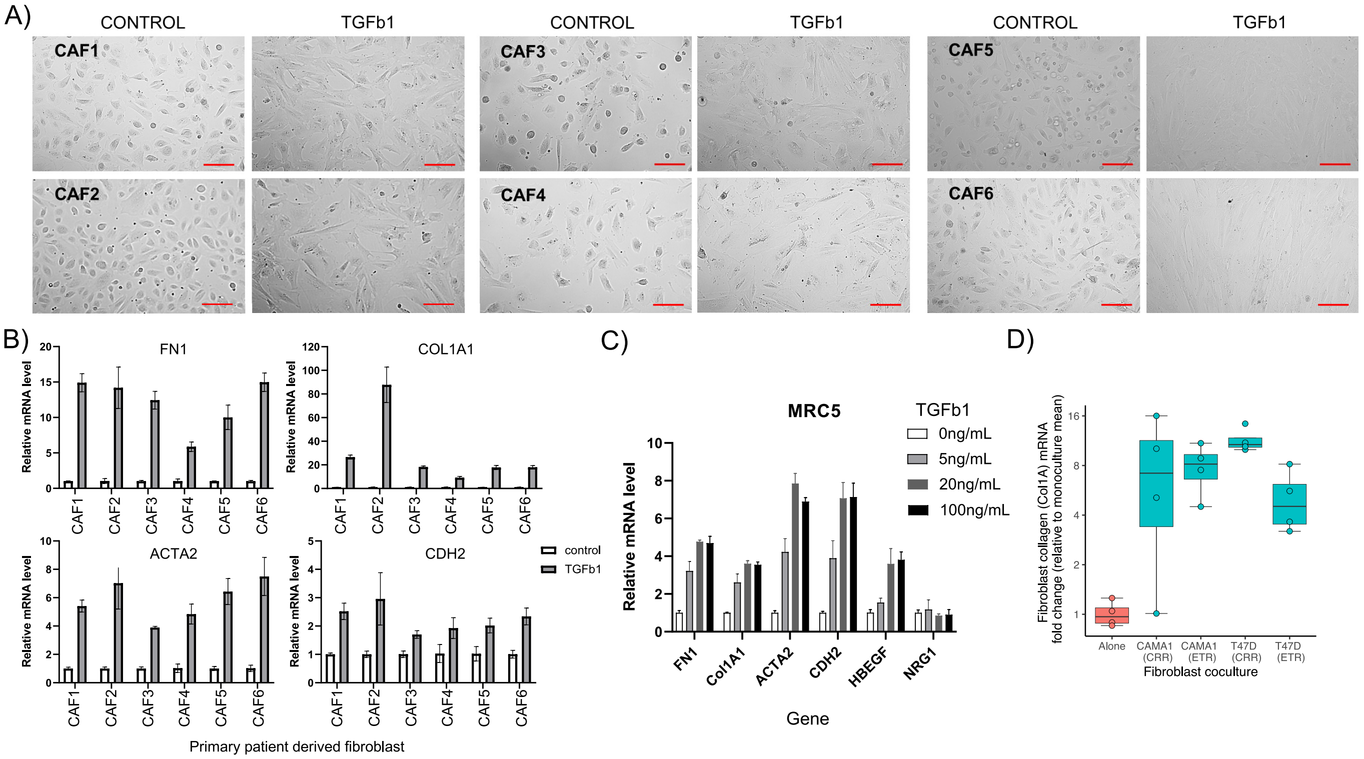

Figure EV4. TGFβ1 or cancer coculture activates fibroblasts differentiation and promotes production of growth factors.

(**A**) Primary patient-derived fibroblast cells ($n = 6$) were isolated from six patients (CAF1-6) and were treated with TGFb1 (20 ng/mL) for 3 days in 2D cultures with medium containing 10% FBS and compared to untreated controls. Images showing morphological change due to TGFb1 treatment compared to control were captured under microscopy (200 x) prior to cell collection for gene expression quantification by qPCR. Scale bars = 50 µm. (**B**) Increased mRNA levels of fibrosis makers (FN1, Col1A1, ACTA2) and EMT maker (CDH2) under TGFb1 treatment (gray) versus control (white) in all ($n = 6$) primary patient-derived fibroblast populations (max CAF p-value: FN1 = 0.0015, COLA1 = 0.0006, ACTA2 = 0.0047, CDH2 = 0.033, HBEGF = 0.0243, NRG1 = 0.0067). Error bars = mean +/− standard error. (**C**) TGFb1 similarly increased mRNA levels of fibrosis makers (FN1, Col1A1, ACTA2), EMT maker (CDH2) and growth factor ligands (HBEGF) in MRC5 fibroblasts treated with TGFb1 (0, 5, 20, 100 ng/mL) in a dose-dependent manner (color) ($n = 3$ replicates per TGFb1 treatment level) (p-value: FN1 = 0.0001, COLA1 = 0.0001, ACTA2 = 0.0001, CDH2 = 0.0001, HBEGF = 0.0004, NRG1 = 0.60). Error bars = mean +/− standard error. (**D**) Cancer cell coculturing stimulates fibroblast differentiation. Fibroblasts (MRC5) increased mesenchymal fibroblast phenotype (measured by Collagen type I alpha 1 (Col1A)) when cocultured with endocrine therapy-resistant (ETR) or combination ribociclib-resistant (CRR) CAMA-1 or T47D cancer cells (blue) compared to when grown alone (red) for 3 days. (ANOVA: Effect of coculture with: T47D (CRR) est = 1.68, se = 0.43, $t = 3.9$, $P = 0.0014$; T47D (ETR) est = 2.02, se = 0.43, $t = 4.7$, $P = 0.0003$; CAMA-1 (CRR) est = 2.43, se = 0.43, $t = 5.7$, $P = 4.6e\text{-}5$; CAMA-1 (ETR) est = 1.60, se = 0.43, $t = 3.65$, $P = 0.002$). Box elements represent median (center line), upper/lower quartiles (hinges), 1.5*interquartile range (whiskers = minima/maxima) for collagen mRNA levels in replicate fibroblast populations (points = replicates) relative to the monoculture mean. Sample size = 20 populations (5 compositions with 4 replicates).

 © The Author(s)

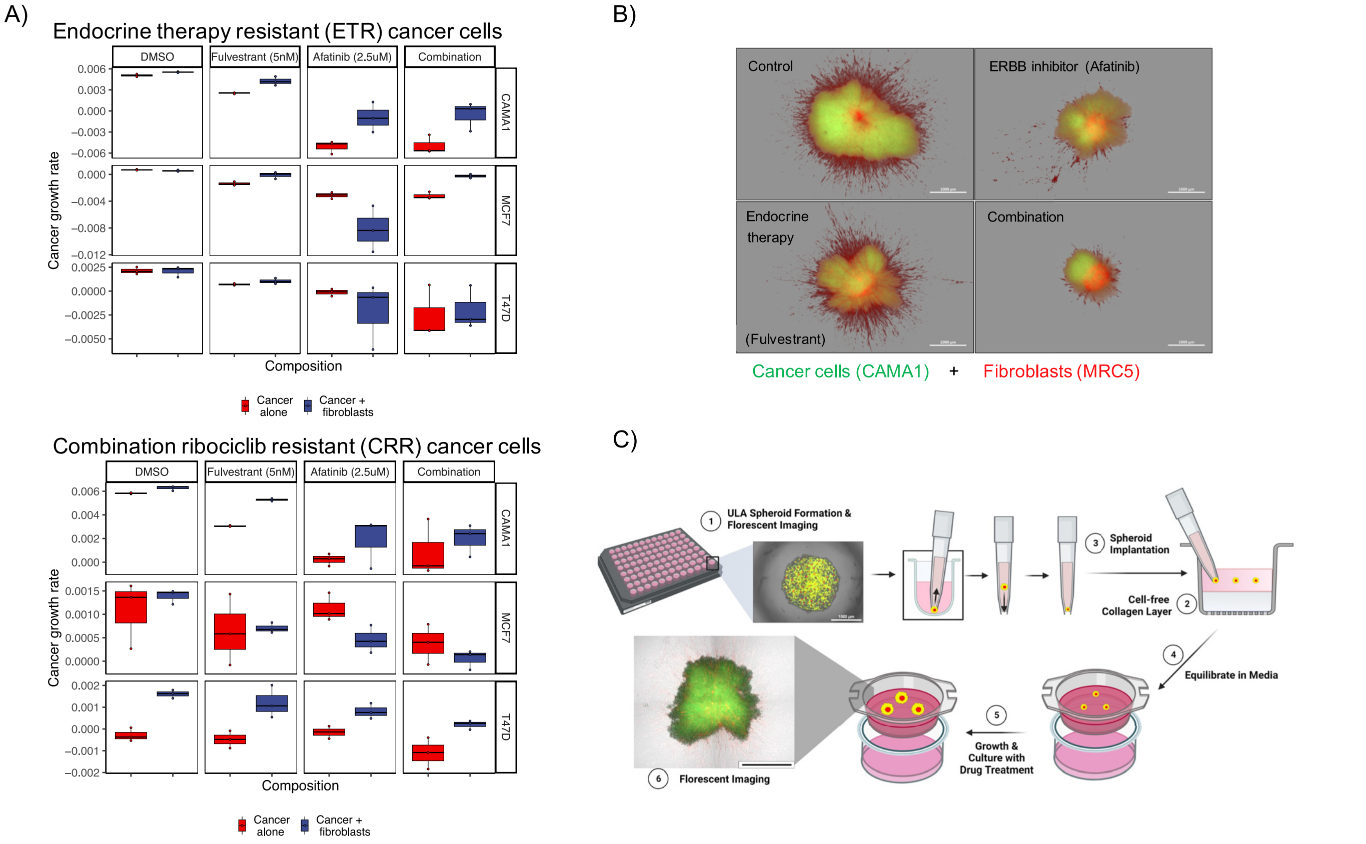

Figure EV5. ERBB pathway inhibition blocks fibroblast facilitation of cancer, control spheroid outgrowth, cancer proliferation and the invasive spiked morphology.

(**A**) ERBB pathway inhibition blocks fibroblast facilitation of cancer promoted by endocrine therapy in both endocrine therapy-resistant (ETR: top) and combination ribociclib-resistant (CRR: bottom) cancer cells. Fibroblast facilitation of cancer population growth blocked by Afatinib (ERBBi). Cancer cells growth rate analysis of CAMA-1, MCF-7 and T47D cancer cells (rows) when monocultured (red) or cocultured with fibroblasts (MRC5) (blue) in 3D spheroids under treatments of afatinib (2.5 μM), fulvestrant (5 nM) or combination (afatinib 2.5 μM + fulvestrant 5 nM) at the indicated concentrations. For all figures, endocrine therapy-resistant cancer cells were labeled with Venus (green), and combination ribociclib-resistant cells were labeled with Cerulean (blue) for CAMA-1, MCF-7 and T47D. MRC5 fibroblasts were seeded together with cancer cells at 1:1 ratio in medium containing 1% FBS. Spheroid Images were captured every 3 or 4 days before medium and drug changes for up to 10 days. Fluorescence intensity was calculated for each image to determine cell abundance and exponential rate of change of cell number measured using least squares. Box elements represent median (center line), upper/lower quartiles (hinges), 1.5*interquartile range (whiskers=minima/maxima) of cancer growth rate (cells/cell/day) across replicate spheroids of a cell line within each treatment. Facilitation of ETR (top panel) and CRR (bottom panel) cancer cell lines (rows; CAMA-1, MCF-7 and T47D) evident by their accelerated growth when cocultured with fibroblasts compared to cancer monocultures under DMSO treatment (first column) and more strongly under fulvestrant treatment (second column). Blockage of facilitation evident under afatinib (third column) and combination treatment (fourth column) by the equivalent or slower growth rate of fibroblast cocultured cancer cells compared to cancer monocultures. Afatinib also slows and often completely prevents the growth of resistant and sensitive cancer cells, especially in combination with fulvestrant. Sample size: $n = 72$ spheroids (3 replicates, 6 cell lines, 2 fibroblast abundances (present/absent), 2 treatments) measured across 5 timepoints. (**B**) Combining endocrine therapy with an ERBB inhibitor controls spheroid outgrowth. Representative images of cancer-fibroblasts spheroid cocultures (SPIKE assay) composed of endocrine therapy-resistant CAMA-1 cancer cells (green: CAMA-1 (ETR)) and fibroblasts (red; MRC5), two weeks after being embedded in matrix and treated with DMSO (control; top left), pan-ERBB inhibitor (afatinib 10 μM; top right), endocrine therapy (fulvestrant 10 nM; bottom left) or combination treatment (bottom right). When cocultured with fibroblasts, control-treated cancer spheroids have a distinct invasive morphology, with cancer cells growing along collagen spikes. Endocrine treated cancer spheroids maintain the spikey morphology, but cancer growth is partially decreased. ERBB inhibition blocks cancer proliferation and the invasive spiked morphology. Combination endocrine and ERBB inhibitor therapy blocks growth and prevents invasive cancer morphology. (**C**) SPIKE assay overview. Cancer-fibroblast coculture spheroids are established for 48 h (1), a transwell culture insert is prepared with an acellular collagen layer (2) followed by spheroid transplanting into an upper collagen layer (3). Spheroids are equilibrated in culture media for 18 h (4) prior to culture media/drug treatment added via the exterior media reservoir (5). Cocultures of cancer cells (green/yellow) and fibroblasts (red) can be monitored by florescent imaging in ULA plates ((1), BioTek Cytation 5, scale = 1,000 μm) or by widefield microscopy (6) (Axio Observer 7, scale = 1000 μm). (Created with Biorender.com).

© The Author(s)
